# α,α-Difluorophosphonohydroxamic Acid Derivatives among the Best Antibacterial Fosmidomycin Analogues

**DOI:** 10.3390/molecules26165111

**Published:** 2021-08-23

**Authors:** Aurore Dreneau, Fanny S. Krebs, Mathilde Munier, Chheng Ngov, Denis Tritsch, Didier Lièvremont, Michel Rohmer, Catherine Grosdemange-Billiard

**Affiliations:** Laboratoire de Chimie et Biochimie de Molécules Bioactives, Université de Strasbourg/CNRS, UMR 7177, Institut Le Bel, 4 Rue Blaise Pascal, 67081 Strasbourg, France; aurore.dreneau@gmail.com (A.D.); krebs.fanny@live.fr (F.S.K.); mathilde.munier01@hotmail.fr (M.M.); chheng.ngov@alsace.cnrs.fr (C.N.); tritsch.denis@neuf.fr (D.T.); didier.lievremont@unistra.fr (D.L.); mirohmer@unistra.fr (M.R.)

**Keywords:** α,α-difluorophosphonate, deoxyxylulose phosphate reductoisomerase, 1-deoxy-dxylulose 5-phosphate reductoisomerase (DXR), antimicrobial, fosmidomycin, isoprenoid biosynthesis, 2-*C*-methyl-derythritol 4-phosphate (MEP) pathway

## Abstract

Three α,α-difluorophosphonate derivatives of fosmidomycin were synthesized from diethyl 1,1-difluorobut-3-enylphosphonate and were evaluated on *Escherichia coli*. Two of them are among the best 1-deoxy-d-xylulose 5-phosphate reductoisomerase inhibitors, with IC_50_ in the nM range, much better than fosmidomycin, the reference compound. They also showed an enhanced antimicrobial activity against *E. coli* on Petri dishes in comparison with the corresponding phosphates and the non-fluorinated phosphonate.

## 1. Introduction

**Scheme 1:**
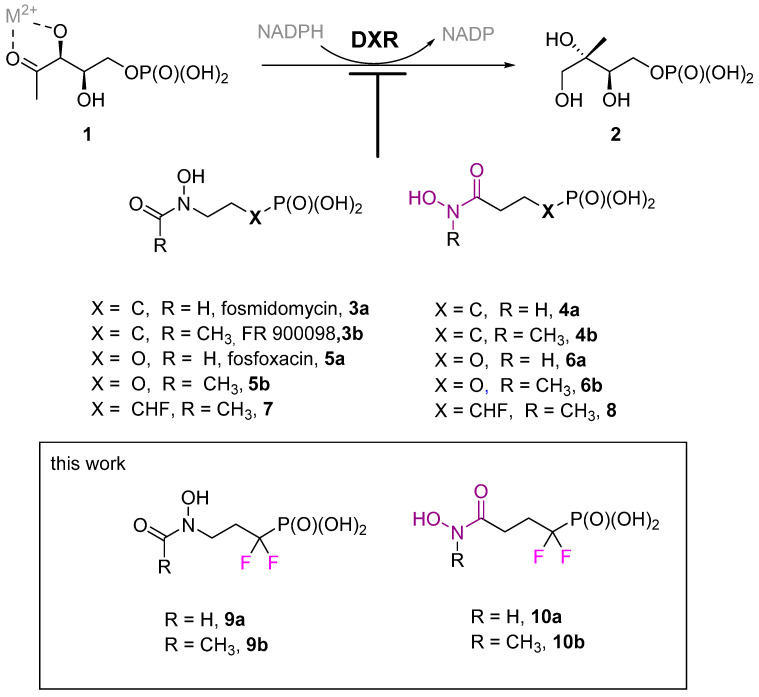
The 1-deoxyxylulose 5-phosphate reductoisomerase (DXR) and its inhibitors.

Antimicrobial resistance affecting anyone in any country is rising to dangerously high levels in all parts of the world and has been recognized as a global health crisis by the United Nations and the World Health Organization (WHO). As a result, the antibiotic treatment of a growing number of infections, e.g., tuberculosis, pneumonia, gonorrhea, and salmonellosis, are becoming less and less effective. In 2017, WHO reported a list of twelve priority pathogens, mostly Gram-negative bacteria belonging to e.g., the *Enterobacteriaceae* or to other groups (e.g., *Acinetobacter baumannii*, *Pseudomonas aeruginosa*…) for which it is urgent to find new treatments [1]. Recently, a study of the European Centre for Disease Prevention and Control (ECDPC) estimated that about 33,000 people died each year from an infection due to antimicrobial-resistant bacteria, frequently while receiving health care i.e., from nosocomial infections [2]. It is therefore crucial and urgent to identify new targets in order to elaborate and develop new drugs. In this respect, the biosynthesis of isopentenyl diphosphate (IPP) and dimethylallyl diphosphate (DMAPP), the two precursors of all isoprenoids, via the 2-*C*-methyl-derythritol 4-phosphate (MEP) pathway is an attractive prospect. In fact, this pathway is essential and present in many Gram-negative and Gram-positive bacteria as well as protozoans, e.g., *Plasmodium* species responsible for malaria [3]. As this pathway is absent in humans, each enzyme is a potential target to elaborate new antimicrobial compounds with expected minimal side effects for the patient. Part of our work on the design of new antimicrobials is based on the inhibition of the second enzyme of the MEP pathway, the 1-deoxy-dxylulose 5-phosphate reductoisomerase (DXR), which catalyzes the conversion of 1-deoxy-d-xylulose 5-phosphate (DXP) **1** into MEP **2** in the presence of a divalent metal ion (Mg^2+^ or Mn^2+^) and NADPH as cofactors (Scheme 1).

Indeed, fosmidomycin **3a** and its *N*-acetyl homologue, FR-900098 **3b**, two natural retrohydroxamate phosphonic acids isolated from *Streptomyces* spp., are selective inhibitors of DXR [4,5]. However, their use in antibiotherapy is limited due to their fast clearance and the rapid emergence of resistance as observed with fosmidomycin [6,7]. In an attempt to improve the efficiency of such inhibitors, numerous analogues of fosmidomycin have been synthesized. From these results, it is clear that neither the retro-hydroxamate chelating moiety nor the phosphonate anchoring group can be replaced without a drastic loss of activity except for the inversion of the retrohydroxamate into a hydroxamate (compounds **4a,b**, Scheme 1) [8] and for the replacement of a phosphonate with a phosphate group (compounds **5a,b** and **6a,b**, Scheme 1) as we previously reported [9].

**Scheme 2:**
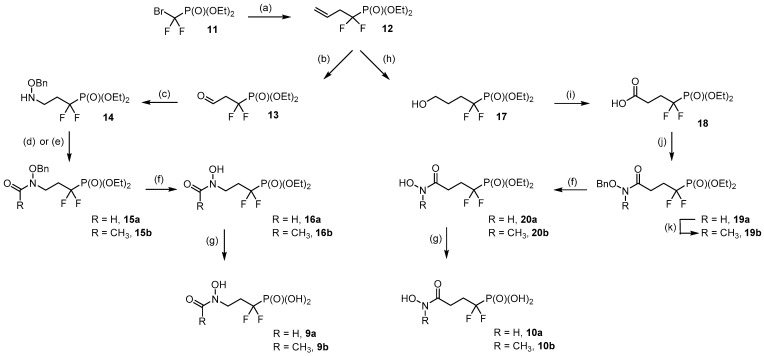
Synthesis of the α,α-difluorophosphonated fosmidomycin derivatives **9** and **10**. Reagents and conditions: (a) Zn, CuBr, then allyl bromide, DMF(89%); (b) (i) O_3,_ MeOH, CH_2_Cl_2_, −78 °C; (ii) Me_2_S, (quantitative); (c) BnONH_2._HCl, NaBH_3_CN, HCl conc, MeOH (35%); (d) HCOOH/Ac_2_O, THF, rt, **15a**, (83%); (d) Ac_2_O, pyridine, rt, **15b**, (80%); (f) H_2_, Pd/C, MeOH, rt, **16a** (74 %), **16b** (80 %), **20a** (41%), **20b** (65%); (g) TMSBr, DCM, 0 °C, then H_2_O, **9b** (quantitative), **10a** (quantitative), **10b** (quantitative); (h,i) BH_3_-THF complex (ii) 3M NaOH, H_2_O_2_ (39%); (i) TEMPO, BAIB, MeCN, H_2_O, (86%); (j) CDI, DCM, 1 h, rt, then BnONH_2_.HCl, Et_3_N, DCM, (77%); (k) MeI, K_2_CO_3_, acetone, 30 min reflux (73%).

Even if the phosphate derivatives (compounds **5a,b**, Scheme 1) were previously shown to be more potent inhibitors of the *Synechocystis* DXR than their phosphonate analogues [10], this conclusion is not valid for all DXRs, as we have shown that they are less efficient against the *E. coli* and *Mycobacterium smegmatis* DXRs [9]. Although a phosphonate group is considered to be isosteric to a phosphate group, some differences such as the pKa values and the C-O-P/C-C-P bond angles might impact their binding in the enzyme active site. Introduction of fluorine atoms in α position of the phosphonate moiety results in significant changes in biological properties and in metabolic stability as compared with the non-fluorinated compounds [11,12,13]. Van Calenbergh et al. reported the synthesis of FR-900098 and its hydroxamic derivative in which the phosphonate has been replaced with a phosphatase-stable α-monofluoromethylenephosphonate **7** and **8** [14]. The racemic mixtures of these synthetic compounds were evaluated in vitro and in vivo for their antimalarial potentials and were shown to be more effective than the reference compounds **3a,b**. The presence of an electron-withdrawing substituent, in particular fluorine, in the α position of the phosphonate moiety, resulted in the decrease of the pKa_2_ of such compounds (from ca. 7.5 to ca. 6.4), almost identical to that of a phosphate group, which is in the dianionic form at the pH of the enzymatic assay, whereas the phosphonate is predominantly in a singly ionized form. By comparison with the α-monofluoromethylene group, which is isoacidic of a phosphate group [15], the α,α-difluoromethylene is an isopolar mimic of the oxygen component of the P-O-C linkage in phosphate [16] and has been used to prepare non-hydrolyzable phosphate analogues of nucleotides [17], phosphatidylinositol [18], glycerol 3-phosphate [11]. In fact, the presence of fluorine atoms able to form fluorine-hydrogen bonds in the DXR active site could affect the binding properties of the parent compounds and also increase their bioavailability [19]. Moreover, the presence of two fluorine atoms might increase the lipophilic properties of the compounds allowing a better cellular uptake [20]. In this context, the synthesis of the protected gem-difluoro FR-900098 derivative **16b** and N-H phosphonohydroxamic acid **10a** (Scheme 2) have been recently reported for a herbicide application [21].

Our investigations are presently orientated toward the synthesis of α,α-difluorophosphonate fosmidomycin derivatives **9a** and **9b** and their analogues **10a** and **10b** to evaluate and determine their effect against *E. coli* DXR in order to develop more potent antimicrobials.

## 2. Results

### 2.1. Chemistry

For the introduction of the fluorine atoms into fosmidomycin **3a** and its analogues **3b** and **4**, we followed the procedure of Shibuya [22] to synthesize diethyl 1,1-difluorobut-3-enylphosphonate **12** [22,23], the parent precursor for all described compounds **9** and **10** (Scheme 2). The key precursor **12** has been prepared by a copper(I) catalyzed coupling reaction of [(diethoxyphosphinyl)difluoromethyl]zinc, formed in situ from the commercially available diethyl bromodifluorophosphonate **11** and Zn dust, with allyl bromide. The synthesis of the diethyl α,α-difluorophosphonate **14** was achieved by previously reported methods [21]. Formylation with the mixed acetyl/formyl anhydride generated in situ from a formic acid and acetic anhydride mixture led to the *N*-formylated compound **15a**, which was obtained as a mixture of conformers due to the restricted rotation around the C-N bond [9,24,25,26] and the large dipole moment of the C-F bond [27]. Acetylation with a mixture of acetic anhydride and pyridine gave the *N*-acetylated analogue **15b** as previously described [21].

The protective benzyl group was removed by catalytic hydrogenolysis with palladium over charcoal at atmospheric pressure and room temperature in methanol giving the deprotected hydroxylamines **16a** and **16b** as a mixture of conformers. Deprotection of the phosphonate group **16** using 10 equivalents of bromotrimethylsilane in DCM following by hydrolysis at room temperature led **9b** as a mixture of conformers. In these conditions, **9a** could not be obtained but led to a deformylated by-product as previously observed [21].

The key step of the synthesis of the α,α-difluorophosphonates **10** was the coupling reaction of the commercially available hydroxylamine hydrochloride with the carboxylic acid **18**. The latter compound was obtained in two steps. Hydroboration-oxidation of the parent precursor **12** in presence of THF complex of BH_3_ and alkaline hydrogen peroxide gave a mixture of the primary and secondary alcohols in a 7/3 ratio, respectively. After purification by flash chromatography, the primary alcohol **17** was oxidized into the carboxylic acid **18** with 2,2,6,6-tetramethyl-1-piperidinyloxyl (TEMPO) in catalytic proportion in presence of [bis(acetoxy)iodo]benzene [28]. Treatment of the carboxylic acid with *O*-benzylhydroxylamine hydrochloride in the presence of 1-(3-dimethyl-aminopropyl)-3-ethylcarbodiimide and 1-hydroxybenzotriazole hydrochloride in DCM gave the hydroxamic acid derivative **19a** [21] as a mixture of conformers. The methyl group was introduced by reaction of **19a** with K_2_CO_3_ in acetone under reflux followed by addition of methyl iodide to give **19b** as a single conformer. Removal of the protective benzyl groups of **19a** and **19b** was achieved by catalytic hydrogenolysis with palladium over charcoal at atmospheric pressure and room temperature leading to **20a** and **20b** as a mixture of conformers. TMSBr-mediated deprotection of the α,α-difluorophosphonate phosphonohydroxamic acid analogues **20** in the same conditions as described for **9b** provided the free phosphonate **10**. Compounds **10a** and **10b** were obtained without further purification as a mixture of conformers.

All α,α-difluorophosphonated compounds **9b** and **10** were tested against His-tagged DXR enzyme of *E. coli* and for growth inhibition against a wild type *E. coli* and fosmidomycin-resistant *E. coli* strain (FosR *E. coli*) as described previously [9].

### 2.2. Biological Activity

#### 2.2.1. Inhibition of *E. coli* H6-DXR with compounds **9b** and **10**

The inhibition potency of α,α-difluorophosphonohydroxamic acid derivatives was characterized by their IC_50_ value that was determined as previously described [8]. Postulating that the α,α-difluorophosphonated analogues act as slow binding inhibitors like fosmidomycin, they were pre-incubated with DXR during 2 min in the presence of NADPH. Residual activity was measured after initiating the enzymatic reaction by addition of DXP. The IC_50_ values are reported in Table 1.

The inhibitory concentration was determined by measuring the phosphorus content of the solution by the method of Lowry and Lopez [29]. *N*-methylated α,α-difluorophosphonates **9b** and **10b** show activity on *E. coli* DXR in the nanomolar concentration range and appear to be 2.5 to 5 times more efficient inhibitors than the parent compound fosmidomycin **3a** (IC_50_ = 9 nM and 17 nM respectively vs. IC_50_ = 42 nM) and slightly less potent inhibitors than FR-900098 **3b** (IC_50_ = 4 nM). The presence of two fluorine atoms in α,α position of the phosphonate group has clearly a positive effect on the affinity of the enzyme for these compounds.

As we have previously reported, except for fosmidomycin **3a**, non-*N*-methylated derivatives are weaker inhibitors than the *N*-methylated homologues [9]. In fact, the N-H α,α-difluorophosphonate **10a** is 280-fold less efficient (IC_50_ = 4600 nM) than its *N*-methylated analogue **10b** and therefore, the poorest inhibitor among all compounds of the non-methylated series. Those results indicated that the replacement of the methylene group or the oxygen atom by a difluoromethylene group enhances the inhibition potency of the *N*-methylated hydroxamic acid derivatives (**4b**, IC_50_ = 48 nM and **6b**, IC_50_ = 46 nM vs. **10b**, IC_50_ = 17 nM) but significantly decreased the inhibition by the N-H analogue **10a**.

#### 2.2.2. Growth Inhibition of a Wild Type *E. coli* and Fosmidomycin Resistant *E. coli* FosR Strain by Compounds **9b**, **10a,b**

The antimicrobial activity of α,α-difluorophosphonate **9a** and **10** was determined using the paper disc diffusion method and was compared with the antimicrobial activity of the non-fluorinated phosphonate and phosphate compounds **3**, **4** et **6**. The diameters of the inhibition zone are given with respect to the amount of inhibitor deposited on the disc (Table 2).

**Figure 1:**
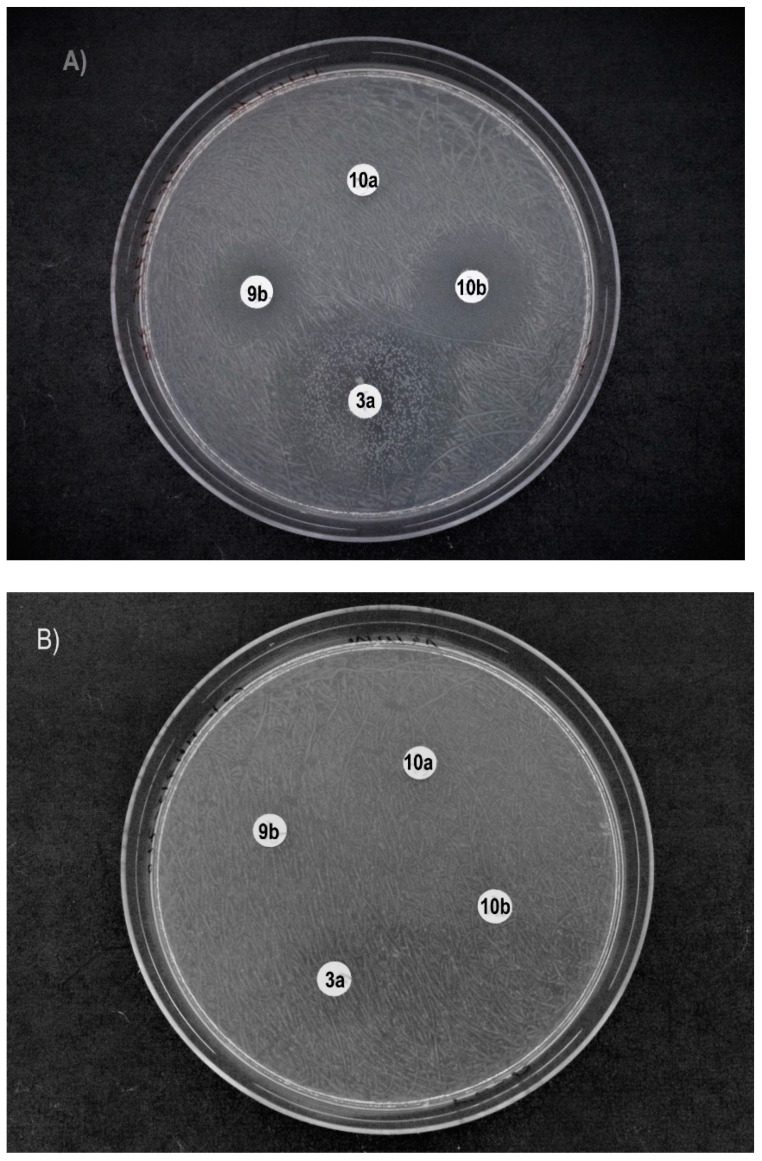
Antimicrobial activity of hydroxamic acids (**9b**, 2 nM), (**10a**, 3 nM) and (**10b**, 1.5 nM) compared to fosmidomycin (**3a**, 2 nM): (**A**) against *E. coli* XL1 Blue; (**B**) against fosmidomycin-resistant strain *E. coli* FosR.

Fosmidomycin, the most efficient growth inhibitors of *E. coli*, was used as a positive control reference. Except for the N-H α,α-difluorophosphonate **10a** where no inhibition was observed, the *N*-methylated derivatives **9b** and **10b** were shown to be quite effective to inhibit bacterial growth (Figure 1A). Similar amounts of **9b** and **10b** had to be added to observe the same growth inhibition zones as those observed for fosmidomycin. Not only the *N*-Me α,α-difluorophosphonates **9b** and **10b** are able to inhibit the DXR in vitro but they are also potent *E. coli* growth inhibitors. Clearly, the presence of the two fluorine atoms in α position of the phosphonate enhances the antimicrobial efficiency of the *N*-methylated phosphonohydroxamic acid. Moreover, we observed in the fosmidomycin **3a** inhibition zone, colonies of tolerant bacteria, which did not appear with the difluoro compounds **9b** and **10b**. These persistent bacteria are known to be able to adapt rapidly to the antibiotic stress, although precise mechanisms are not fully understood [30]. Interestingly, α,α-difluoro compounds **9b** and **10b** eliminated this survival ability of the *E. coli* population.

All DXR α,α-difluorophosphonate inhibitors were tested on a fosmidomycin resistant strain of *E. coli* (FosR), but none of the compounds was able to affect bacterial growth (Figure 1B).

## 3. Discussion

Among a large variety of synthetic analogues of natural phosphono- and phospho- retrohydroxamic acids, e.g., fosmidomycin **3a**, FR-900098 **3b** and fosfoxacin **5a**, only the *N*-H gem-difluoro **10a** have been reported and evaluated as herbicide [21]. However, Van Calenbergh and co-workers observed that the racemic monofluoro analogues **7** and **8** were more active than the parent compounds fosmidomycin and **4b** against intraerythrocytic forms of *Plasmodium falciparum* (K1 strain). Interestingly, none of them have been tested against bacteria [14]. Such promising results prompted us to evaluate the efficiency of α,α-difluorophosphonate analogues of FR-900098 and its phosphonohydroxamic acid derivatives **10a** and **10b** against *E. coli*. Except for the N-H α,α-difluorophosphononate **10a**, the *N*-methylated CF_2_-phosphonates **9b** and **10b** exhibited stronger inhibition activity than that of the reference compound fosmidomycin against *E. coli* DXR. With an IC_50_ value of 17 nM, the *N*-methyl α,α-difluorophosphono hydroxamic acid **10b** represents the most powerful inhibitor compared to the non-fluorinated **4b** (IC_50_ = 48 nM) and phosphate **6b** (IC_50_ = 46 nM) analogues. Even if the activity of the CF_2_-FR-900098 derivative **9b** (IC_50_ = 9 nM) is two-fold less active than the parent compound **3b** (IC_50_ = 4 nM), it remains a better inhibitor of the *E. coli* DXR than its phosphate derivative **5b** (IC_50_ = 77 nM).

The introduction of the two electron-withdrawing fluorine atoms on the α-methylene group of the phosphonate significantly decreases the pKa2 from 7.6 for the phosphonate to ca. 5.4. The α,α-difluorophosphonates should therefore be in the dianionic form at the pH of the enzyme assay much like a phosphate (pKa2 = 6.4), thereby favoring a more efficient binding than the phosphonate, which is mostly in the singly ionized form. In addition, the dihedral C-CF_2_-P angle (116.1°), wider than the C-CH_2_-P (112.1°), closely resembles that of the phosphate C-O-P angle of 118.7° [12]. The α,α-difluorophosphonates were, however, shown to be better inhibitors than the phosphate analogues, resulting in a better setting in the *E. coli* DXR active site. The performance of the α,α-difluorophosphonate analogues cannot thus be attributed to ionization or geometry and could be mostly due to favorable modifications of the electrostatic and van der Waals interactions, leading to an increase of the affinity for those inhibitors.

Compared to the *N*-methylated phosphonate and phosphate, the CF_2_-phosphonates **9b** and **10b** inhibited efficiently the growth of *E. coli* at doses similar to those of fosmidomycin and FR-900098, making them powerful promising antimicrobials. It is generally accepted that introduction of fluorine atoms enhances the lipophilicity of the compounds, which might facilitate a passive diffusion across the cell membrane of the α,α-difluorophosphonate inhibitors. However, we recently reported that, except for the phosphonate **4b**, all phosphate compounds **5** and **6** penetrated into the bacteria via the same transporters as those involved in the transport of e.g., the glycerol 3-phosphate and the hexose 6-phosphate like fosmidomycin and FR-900098 [9]. No growth inhibition by the α,α-difluoro phosphonates **9b** and **10** against fosmidomycin resistant strain *E. coli* (FosR), in which the GlpT/UhpT transporters are therefore inoperative, was observed implying that these inhibitors penetrate into the bacteria via these transporters (Figure 1B).

In summary, three α,α-difluorophosphonate derivatives of fosmidomycin **3a** were synthesized and were shown, except for the N-H difluoro compound **10a**, to be powerful inhibitors against *E. coli* DXR. Among the series of hydroxamic acids derivatives, the inhibitor **10b** surpasses the phosphonate and phosphate analogues in the inhibition of DXR enzyme as well as in the antimicrobial activity. For *N*-Me difluorophosphonate **9b** and **10b**, there is a direct relation between the capacity to inhibit the DXR and the bacterial growth. An important outcome of this study is that the introduction of two fluorine atoms on the α-methylene group, favors the inhibition on the DXR and enhances the antimicrobial activity in comparison with the phosphates and the non-fluorinated phosphonates.

## 4. Materials and Methods

### 4.1. Chemistry

#### 4.1.1. General Methods

All non-aqueous reactions were run in oven-dried glassware under an argon atmosphere, using dry solvents. Commercial grade reagents were purchased from Sigma-Aldrich, Acros Organics or Thermo Fischer Scientific and used without further purification. Petroleum ether (PE) 40–60 °C (Sigma-Aldrich, St-Louis, MO, USA) was used for chromatography. Flash chromatography was performed on silica gel 60 230–400 mesh with the solvent system as indicated. TLC plates were revealed under UV light (254 nm) and/or by spraying with an ethanolic solution of phosphomolybdic acid (20%) or an ethanolic solution of potassium permanganate followed by heating. The NMR spectra (Supplementary Materials) were recorded on a Bruker Avance 300 (^1^H-NMR: 300 MHz; ^13^C-NMR, 75.5 MHz; ^31^P-NMR 121.5 MHz; ^19^F-NMR 282.4 MHz), a Bruker Avance 400 (^1^H NMR: 400 MHz; ^13^C NMR: 100 MHz; ^31^P NMR: 162 MHz) or a Bruker Avance 500 (^1^H-NMR: 500 MHz; ^13^C-NMR, 125.8 MHz). ^1^H-NMR experiments were performed in CDCl_3_ with CHCl_3_ (δ = 7.26 ppm) or CD_3_OD with CD_2_HOD (δ = 3.31 ppm) as internal references. ^13^C-NMR experiments were performed in CDCl_3_ with CDCl_3_ (δ = 77.23 ppm) or in CD_3_OD with CD_2_HOD (δ = 49.0 ppm) as internal references. For ^31^P-NMR and ^19^F-NMR references, the spectrometer had external references, corresponding to 80% phosphoric acid in D_2_O (δ = 0 ppm) and to 0.05% α,α,α-trifluorotoluene in CDCl_3_ (δ = −62.75 ppm). Chemical shifts are expressed in ppm and signal multiplicities are described using the following abbreviations: s for singlet, d for doublet, t for triplet, q for quartet, p for quintet and m for multiplet. In the presence of conformers, signals were differentiated by a * sign added to the assignments. Negative or positive-mode electrospray MS were performed on a Bruker Daltonics microTOF spectrometer (Bruker Daltonik GmbH, Bremen, Germany) equipped with an orthogonal electrospray (ESI) interface. Calibration was performed using a solution of 10 mM sodium formate. Sample solutions were introduced into the spectrometer source with a syringe pump (Harvard type 55 1111: Harvard Apparatus Inc., South Natick, MA, USA) with a flow rate of 5 μL min^−1^.

#### 4.1.2. Synthesis of α,α-Difluorophosphonate Derivatives

*Synthesis of the intermediate diethyl 1,1-difluorobut-3-enylphosphonate* (**12**) [31]. A solution of BrZnCF_2_P(O)(OEt)_2_ was prepared from diethyl (bromodifluoromethyl)phosphonate (2.67 g, 10 mmol) and Zn dust (0.65 g, 10 mmol) in DMF (18 mL). The reaction was stirred for 3 h at room temperature. CuBr (1.43 g, 10 mmol) was added, and the reaction mixture was stirred for 30 min before the addition of allyl bromide (0.43 mL, 5 mmol). The reaction mixture was stirred overnight, quenched with a 10% aqueous solution of HCl (10 mL), filtered through celite and extracted with Et_2_O (3 × 15 mL). The organic layers were combined, washed with a saturated solution of NaHCO_3_ then with brine, dried over anhydrous Na_2_SO_4_ and evaporated to dryness. The product is obtained after purification by column chromatography (EtOAc/PE 3:7 to 1:1) as a colorless oil (1.02 g, 89% yield). *R_f_* = 0.54 (EtOAc/PE 1:1); ^1^H NMR (300 MHz, CDCl_3_): δ = 1.37 (6H, t, *J* = 7.1 Hz), 2.73–2.93 (2H, m), 4.26 (4H, dq, *J*_HH_ = *J*_PH_ = 7.1 Hz), 5.22–5.31 (2H, m), 5.73–5.92 (1H, m); ^13^C NMR (75.5 MHz, CDCl_3_): δ = 16.5 (CH_3_ × 2, d, *J*_CP_ = 5.6 Hz), 38.8 (CH_2_, dt, *J*_CF_ = 21.5 Hz, *J*_CP_ = 15.1 Hz), 64.5 (CH_2_ × 2, d, *J*_CP_ = 6.8 Hz), 119.7 (CF_2_, dt, *J*_CF_ = 260.0 Hz, *J*_CP_ = 215.1 Hz), 121.4 (CH_2_), 127.1 (CH, dt, *J*_CF_ = 11.4 Hz, *J*_CP_ = 5.6 Hz); ^19^F NMR (282 MHz, CDCl_3_): δ = −111.26 (d, *J*_FP_ = 107.5 Hz); ^31^P NMR (121 MHz, CDCl_3_): δ = 6.9 (t, *J*_PF_ = 107.5 Hz).

#### 4.1.3. Synthesis of the Target Compounds (**9**)

*Diethyl (1,1-difluoro-3-oxopropyl)phosphonate* (**13**) [32]. Ozone was bubbled through a solution of diethyl 1,1-difluorobut-3-enylphosphonate **12** (2.28 g, 10 mmol) in DCM/MeOH (50 mL, 4:1) at −78 °C until the solution turned blue (5 min). Nitrogen was bubbled through the solution until the blue color disappeared (removal of excess ozone). Dimethylsulfide (1.8 mL, 24.5 mmol) was added at −78 °C and the solution was allowed to warm up to room temperature. Solvents and excess of Me_2_S were removed under reduced pressure, giving **13** as a colorless oil, which was immediately used without further purification for the next reaction. *R_f_* = 0.46 (PE/EtOAc 1:1); ^1^H NMR (300 MHz, CDCl_3_): δ = 1.36 (6H, td, *J*_HH_ = 7.1 Hz, *J*_HP_ = 0.6 Hz), 3.06 (2H, tdd, *J*_HF_ = 19.1 Hz, *J*_HP_ = 7.0 Hz, *J*_HH_ = 2.5 Hz), 4.27 (4H, dq, *J*_HP_ = 8.2 Hz, *J*_HH_ = 7.1 Hz), 9.77 (1H, t, *J* = 2.5 Hz).

*Diethyl (3-((benzyloxy)amino)-1,1-difluoropropyl)phosphonate* (**14**) [21]. Diethyl (1,1-difluoro-3-oxopropyl)phosphonate **13** (2.3 g, 10 mmol) and *N*-hydroxybenzylamine hydrochloride (1.6 g, 10 mmol) in 8 mL MeOH were stirred for 1 h at room temperature. The solution was diluted with more MeOH (142 mL), and sodium cyanoborohydride (1.89 g, 30 mmol) was added portion wise over 30 min. The reaction mixture was cooled to 0 °C before the dropwise addition of HCl 37% in water (10 mL, 100 mmol) over 40 min. Sodium cyanoborohydride (0.44 g) was then added at room temperature and the mixture left to stir for 2 h. The solution was evaporated and treated with aqueous KOH (10%) to obtain a basic pH. The product was extracted with EtOAc (3 × 70 mL), and the combined organic layers were dried over Na_2_SO_4_ and evaporated to dryness under reduced pressure. **14** (1.19 g, 35% yield) was obtained as a transparent oil after purification by chromatography column on silica gel with PE/EtOAc (100:0 to 50:50) as eluent. R_f_ = 0.54 (EtOAc/PE 7:3); ^1^H NMR (500 MHz, CDCl_3_): δ = 1.37 (6H, t, *J* = 7.1 Hz), 2.36 (2H, tq, *J*_HF_ = 20.2 Hz, *J*_HH_ = *J*_HP_ = 6.6 Hz), 3.18–3.25 (2H, t, *J* = 7.1 Hz), 4.22–4.31 (4H, m), 4.70 (2H, s), 5.76 (1H, br s), 7.27–7.39 (5H, m); ^13^C NMR (125 MHz, CDCl_3_): δ = 16.4 (CH_3_ × 2, d, *J*_CP_ = 5.1 Hz), 32.2 (CH_2_CF_2_, td, *J*_CF_ = 20.4 Hz, *J*_CP_ = 14.4 Hz), 44.4 (CH_2_N, q, *J*_CF_ = 5.1 Hz), 64.6 (CH_2_ × 2, d, *J*_CP_ = 6.7 Hz), 76.2 (CH_2_Ph), 120.5 (CF_2_, td, *J*_CF_ = 257.8 Hz, *J*_CP_ = 214.7 Hz), 127.9 (CH_ar_), 128.4 (CH_ar_ × 2), 128.4 (CH_ar_ × 2), 137.7 (C_ar_); ^19^F NMR (282 MHz, CDCl_3_): δ = −111.08 (d, J_FP_ = 107.9 Hz); ^31^P NMR (121 MHz, CDCl_3_): δ = 7.00 (t, *J*_PF_ = 107.3 Hz).

*Diethyl (3-(N-(benzyloxy)formamido)-1,1-difluoropropyl)phosphonate* (**15a**). Formic acid (2.8 mL, 74.1 mmol) and acetic anhydride (1.4 mL, 14.8 mmol) were stirred for 30 min at room temperature. The solution was cooled to 0 °C then diethyl (3-((benzyloxy)amino)-1,1-difluoropropyl)phosphonate **14** (500 mg, 1.5 mmol) dissolved in anhydrous THF (1.5 mL) was added dropwise. The reaction mixture was stirred for 10 min at 0 °C and overnight at room temperature. EtOAc (15 mL) was added and the resulting organic layer washed with water (2 × 10 mL) and aqueous KOH (0.1 M, 10 mL), dried over Na_2_SO_4_ and evaporated to dryness under reduced pressure. A purification by flash chromatography on silica gel with EtOAc/PE 7:3 as eluent gave **15a** as a light yellow oil (450 mg, 83% yield) and as a mixture of two conformers in a 2:8 ratio, respectively. R_f_ = 0.54 (EtOAc/PE 7:3); ^1^H NMR (300 MHz, CDCl_3_): δ = 1.37 (6H, t, *J* = 7.2 Hz), 2.29–2.52 (2H, m), 3.48–3.70 (2/10 of 2H, s), 3.76–3.94 (8/10 of 2H, s), 4.20–4.33 (4H, m), 4.86 (2H, s), 7.27–7.39 (5H, m), 8.16 (1H, s); ^13^C NMR (125 MHz, CDCl_3_): δ = 16.4 (CH_3_ × 2, d, J_CP_ = 5.4 Hz), 30.6–31.4 (CH_2_CF_2_, m), 37.2 (CH_2_N), 41.8 (CH_2_N*), 64.72 (CH_2_ × 2, d, *J*_CP_ = 6.7 Hz), 77.8 (CH_2_Ph), 119.6 (CF_2_, td, *J*_CF_ = 258.6 Hz, *J*_CP_ = 215.1 Hz), 128.8 (CH_ar_), 129.2 (CH_ar_*), 129.5 (CH_ar_), 134.1 (C_ar_), 134.6 (C_ar_*), 158.2 (CO*), 163.3 (CO); ^19^F NMR (282 MHz, CDCl_3_): δ = −113.0 (d, *J*_FP_ = 121.2 Hz), −113.4 (d, *J*_FP_ = 106.2 Hz); ^31^P NMR (121 MHz, CDCl_3_): δ = 6.38 (t, *J*_PF_ = 105.8 Hz); HRMS (ESI^+^) *m*/*z* calcd for C_15_H_22_F_2_NNaO_5_P [M + Na]^+^ 388.1096, found 388.1099.

*Diethyl (3-(N-(benzyloxy)acetamido)-1,1-difluoropropyl)phosphonate* (**15b**) [21]. Anhydrous pyridine (0.4 mL, 4.4 mmol) was added dropwise to a solution of diethyl (3-((benzyloxy)amino)-1,1-difluoropropyl)phosphonate **14** (500 mg, 1.5 mmol) in acetic anhydride (6 mL, 63.5 mmol). The reaction mixture was stirred overnight at room temperature then evaporated to dryness under reduced pressure. A purification by flash chromatography on silica gel with EtOAc/PE 7:3 as eluent gave **15b** as a transparent oil (450 mg, 80% yield) and as a single conformer. R_f_ = 0.55 (EtOAc/PE 7:3); ^1^H NMR (300 MHz, CDCl_3_): δ = 1.37 (6H, t, *J* = 7.6 H), 2.07 (3H, s), 2.29–2.53 (2H, m), 3.87–3.95 (2H, m), 4.26 (4H, pseudo p, *J*_HH_ = *J*_HP_ = 7.2 Hz), 4.83 (2H, s), 7.30–7.43 (5H, m); ^13^C NMR (125 MHz, CDCl_3_): δ = 16.4 (CH_3_ × 2, d, J_CP_ = 5.4 Hz), 20.6 (CH_3_CO), 30.5–31.1 (CH_2_CF_2_, m), 38.5 (CH_2_N), 64.7 (CH_2_ × 2, d, J_CP_ = 6.8 Hz), 76.5 (OCH_2_Ph), 119.8 (CF_2_, td, *J*_CF_ = 259.9 Hz, *J*_CP_ = 216.3 Hz), 128.8 (CH_ar_ × 2), 129.1 (CH_ar_), 129.3 (CH_ar_ × 2), 134.2 (C_ar_), 172.9 (CO); ^19^F NMR (282 MHz, CDCl_3_): δ = −112.33 (d, *J*_FP_ = 106.7 Hz); ^31^P NMR (121 MHz, CDCl_3_): δ = 6.38 (t, *J*_PF_ = 106.7 Hz).

*Diethyl (1,1-difluoro-3-(N-hydroxyformamido)propyl)phosphonate* (**16a**). Diethyl (3-(*N*-(benzyloxy)formamido)-1,1-difluoropropyl)phosphonate **15a** (438 mg, 1.2 mmol) and palladium on charcoal (45 mg, 10%) were introduced in a 2-neck-flask with a three-way tap under a nitrogen atmosphere. After the addition of HPLC grade MeOH (30 mL), the reaction mixture was degassed three times then left to stir for 24 h under hydrogen at atmospheric pressure. The solution was filtered over celite and evaporated to dryness under reduced pressure. A purification by chromatography column with EtOAc as eluent gave **16a** as an orange oil (245 mg, 74% yield) and as a mixture of two conformers in a 1:1 ratio. R_f_ = 0.39 (EtOAc); ^1^H NMR (500 MHz, CDCl_3_): δ = 1.38 (6H, t, *J* = 7.1 Hz), 2.37–2.58 (2H, m), 3.80–3.88 (2H, m), 4.28 (4H, pseudo p, *J*_HH_ = *J*_HP_ = 7.4 Hz), 7.91 (1/2 of 1H, s), 8.35 (1/2 of 1H, s); ^13^C NMR (125 MHz, CDCl_3_): δ = 16.3 (CH_3_ × 2, d, *J*_CP_ = 5.4 Hz), 16.4 (CH_3_ × 2, d, *J* = 5.4 Hz), 31.6 (CH_2_CF_2_, dt, *J*_CF_ = 20.7 Hz, *J*_CP_ = 14.4 Hz), 32.7 (CH_2_CF_2_*, dt, *J*_CF_ = 20.7 Hz, *J*_CP_ = 14.4 Hz), 41.6 (CH_2_N, q, *J*_CF_ = 6.1 Hz), 42.5 (CH_2_N*, q, *J*_CF_ = 6.1 Hz), 65.0 (CH_2_O × 2, d, *J*_CP_ = 7.0 Hz), 65.6 (CH_2_O* × 2, d, *J*_CP_ = 7.0 Hz), 119.6 (CF_2_, td, *J*_CF_ = 259.3 Hz, *J*_CP_ = 211.7 Hz), 119.9 (CF_2_*, td, *J*_CF_ = 259.3 Hz, *J*_CP_ = 211.7 Hz), 156.3 (CO*), 163.1 (CO); ^19^F NMR (282 MHz, CDCl_3_): δ = −112.70 (d, J_FP_ = 105.0 Hz), −109.12 (d, *J*_FP_ = 107.7 Hz); ^31^P NMR (121 MHz, CDCl_3_) δ 6.14 (t, *J*_PF_ = 104.7 Hz), 7.39 (t, *J*_PF_ = 107.4 Hz); HRMS (ESI^+^) *m*/*z* calcd for C_8_H_16_F_2_NNaO_5_P [M + Na]^+^ 298.0626, found 298.0625.

*Diethyl (1,1-difluoro-3-(N-hydroxyacetamido)propyl)phosphonate* (**16b**) [21]. Diethyl (3-(*N*-(benzyloxy)acetamido)-1,1-difluoropropyl)phosphonate **15b** (450 mg, 1.2 mmol) and palladium on charcoal (45 mg, 10%) were introduced in a 2-neck-flask with a three-way tap under a nitrogen atmosphere. After the addition of HPLC grade MeOH (30 mL), the reaction mixture was degassed three times then left to stir for 24 h under a hydrogen atmosphere. The solution was filtered over a pad of celite and evaporated to dryness under reduced pressure. A purification by chromatography column with EtOAc as eluent gave **16b** as a light yellow oil (280 mg, 80% yield) and as a mixture of two conformers in a 2:8 ratio respectively. R_f_ = 0.41 (EtOAc); ^1^H NMR (500 MHz, CDCl_3_): δ = 1.38 (6H, t, *J* = 7.1 Hz), 2.13 (3H, s), 2.37–2.55 (2H, m), 3.82–3.90 (8/10 of 2H, t, *J* = 5.9 Hz), 3.91–3.99 (2/10 of 2H, m), 4.27 (4H, pseudo p, *J*_HH_ = *J*_HP_ = 6.8 Hz); ^13^C NMR (125 MHz, CDCl_3_): δ = 16.3 (CH_3_ × 2, d, *J*_CP_ = 5.5 Hz), 18.3 (CH_3_CO*), 20.4 (CH_3_CO), 31.2–31.7 (CH_2_CF_2_*, m), 33.3 (CH_2_CF_2_, dt, *J*_CF_ = 22.0 Hz, *J*_CP_ = 14.7 Hz), 41.9–42.2 (CH_2_N*, m), 43.3–43.6 (CH_2_N, m), 64.7–65.0 (CH_2_O* × 2, m), 65.50 (CH_2_O × 2, d, *J*_CP_ = 7.3 Hz), 120.2 (CF_2_, td, *J*_CF_ = 258.7 Hz, *J*_CP_ = 210.7 Hz), 164.8 (CO*), 172.6 (CO); ^19^F NMR (282 MHz, CDCl_3_): δ = −113.49 (d, J_FP_ = 105.1 Hz), −108.2 (d, *J*_FP_ = 108.5 Hz); ^31^P NMR (121 MHz, CDCl_3_): δ = 6.17 (t, *J*_PF_ = 106.1 Hz), 7.57 (t, *J*_PF_ = 108.1 Hz).

*(1,1-Difluoro-3-(N-hydroxyacetamido)propyl)phosphonic acid* (**9b**). A solution of diethyl (1,1-difluoro-3-(*N*-hydroxyacetamido)propyl)phosphonate **16b** (25 mg, 86 µmol) dissolved in DCM (0.32 mL) was cooled to 0 °C. TMSBr (0.11 mL, 0.9 mmol) was added dropwise at 0 °C then the reaction mixture was stirred overnight in the dark at room temperature. DCM and excess of TMSBr were evaporated under reduced pressure and the intermediate orange oil was treated with water (0.5 mL, 27.8 mmol) for 1.5 h. Removal of water under vacuum afforded **9b** as an orange oil (20 mg, quant. yield) and as a mixture of conformers. *R_f_* = 0.14 (EtOAc); ^1^H NMR (400 MHz, CD_3_OD): δ = 2.02 (5/10 of 3H, s), 2.10 (5/10 of 3H, s), 2.34–2.46 (4/10 of 2H, m), 2.52–2.64 (6/10 of 2H, m), 3.53 (5/10 of 2H, t, *J* = 7.6 Hz), 3.89 (4/10 of 2H, t, *J* = 7.6 Hz), 4.10–4.26 (1/10 of 2H, m); ^13^C NMR (125 MHz, CD_3_OD): δ = 20.2 (CH_3_CO*), 20.6 (CH_3_CO), 29.6 (CH_2_CF_2_, td, *J*_CF_ = 22.3 Hz, *J*_CP_ = 15.7 Hz), 32.0 (CH_2_CF_2_*, td, *J*_CF_ = 20.2 Hz, *J*_CP_ = 14.5 Hz), 42.2–42.5 (CH_2_N*, m), 45.6–45.8 (CH_2_N, m), 120.6 (CF_2_, td, *J*_CF_ = 258.9 Hz, *J*_CP_ = 210.2 Hz), 173.4 (CO), 173.9 (CO*); ^19^F NMR (282 MHz, CD_3_OD): δ = −115.5 (d, *J*_FP_ = 100.3 Hz), −115.7 (d, *J*_FP_ = 99.4 Hz), −116.3 (d, *J*_FP_ = 103.7 Hz); ^31^P NMR (162 MHz, CD_3_OD): δ = 5.00 (t, *J*_PF_ = 103.8 Hz), 3.96 (t, *J*_PF_ = 100.1 Hz), 3.78 (t, *J*_PF_ = 99.6 Hz); HRMS (ESI^−^) *m*/*z* calcd for C_5_H_10_F_2_NO_5_P [M − H]^−^ 232.0192, found 232.0181.

#### 4.1.4. Synthesis of the Target Compounds (**10**)

*Diethyl 1,1-difluorobutan-4-ol phosphonate* (**17**). A 1 M solution of borane in THF (32 mL, 32 mmol) was added dropwise to a stirred solution of diethyl 1,1-difluorobut-3-enylphosphonate **12** (1.84 g, 8 mmol) in THF (20 mL) at 0 °C. After 30 min, the reaction mixture was allowed to warm up at room temperature for 4 h. More borane solution (16 mL) was added dropwise at 0 °C and the mixture was stirred overnight at room temperature. Then, methanol (10 mL), a 3 M solution of NaOH in water (4 mL) and a 30% aqueous solution of H_2_O_2_ (4 mL) were successively added. The mixture was heated at 50 °C for 1 h, quenched with brine (30 mL) and extracted with chloroform (3 × 20 mL). The organic layers were combined, dried over anhydrous Na_2_SO_4_ and evaporated to dryness. Purification by column chromatography (PE/EtOAc 3:2 to 100% EtOAc) yielded **17** as a colorless oil (770 mg, 39% yield). R_f_ = 0.29 (EtOAc/PE 7:3); ^1^H NMR (300 MHz, CDCl_3_): δ = 1.37 (6H, td, *J*_HH_ = 7.1 Hz, *J*_PH_ = 0.6 Hz), 1.62 (1H, br s, OH), 1.78–1.87 (2H, m), 2.06–2.26 (2H, m), 3.67 (2H, t, *J* = 6.2 Hz), 4.26 (4H, dq, *J*_PH_ = 7.9 Hz, *J*_HH_ = 7.1 Hz); ^13^C NMR (125 MHz, CDCl_3_): δ = 16.4 (CH_3_, d, *J*_CP_ = 5.5 Hz), 24.0 (CH_2_, dt, *J*_CF_ = 4.9 Hz, *J*_CP_ = 4.4 Hz), 30.5 (CH_2,_ td, *J*_CF_ = 21.1 Hz, *J*_CP_ = 14.9 Hz), 61.9 (CH_2_OH), 64.5 (CH_2_ × 2_,_ d, *J*_CP_ = 6.8 Hz), 121.0 (CF_2_, td, *J*_CF_ = 259.3 Hz, *J*_CP_ = 215.7 Hz); ^19^F NMR (282 MHz, CDCl_3_): δ = 112.7 (d, *J*_FP_ = 109.3 Hz); ^31^P NMR (121 MHz, CDCl_3_): δ = 7.49 (t, *J*_PF_ = 109.3 Hz).

*Difluoro-4-(diethyl phosphonate) butanoic acid* (**18**) [21]. To a stirred solution of **17** (400 mg, 1.6 mmol) in MeCN (4 mL) were successively added TEMPO (51 mg, 0.3 mmol), BAIB (1.151 g, 3.6 mmol) and water (4 mL). The reaction mixture was stirred overnight then concentrated under reduced pressure. An aqueous saturated solution of NaHCO_3_ was added to reach a pH > 8. The solution was extracted with DCM (3 × 15 mL) to remove excess of starting materials. A 10% HCl solution was added to the aqueous layer until pH < 5. Then the solution was extracted with DCM (3 × 15mL), the organic layer was dried over anhydrous Na_2_SO_4_ and evaporated to dryness. The product was not further purified and obtained as a light yellow oil (420 mg, 99% yield). R_f_ = 0.26 (EtOAc/PE 7:3); ^1^H NMR (500 MHz, CDCl_3_): δ = 1.38 (6H, t, *J* = 7.1 Hz), 2.35–2.51 (2H, m), 2.63–2.70 (2H, m), 4.28–4.31 (4H, pseudo p, *J*_HH_ = *J*_PH_ = 7.2 Hz), 9.61 (1H, br s); ^13^C NMR (125 MHz, CDCl_3_): δ = 16.5 (CH_3_ × 2, d, *J*_CP_ = 5.5 Hz), 25.8 (CH_2_CF_2_, q, *J*_CP_ = 5.4 Hz), 29.1 (CH_2_CO, dt, *J*_CF_ = 21.2 Hz, *J*_CP_ = 15.9 Hz), 64.8 (CH_2_ × 2, d, *J*_CP_ = 6.9 Hz), 119.8 (CF_2_, dt, *J*_CF_ = 259.8 Hz, *J*_CP_ = 217.2 Hz), 176.5 (CO); ^19^F NMR (282 MHz, CDCl_3_): δ = −112.75 (d, *J*_FP_ = 107.6 Hz); ^31^P NMR (121 MHz, CDCl_3_): δ = 6.48 (t, *J*_PF_ = 107.5 Hz.

*Diethyl 4-(N-benzyloxy)-amino-1,1-difluoro-4-oxobutyl phosphonate* (**19a**) [21]. CDI (0.528 g, 3.2 mmol) and BnONH_2_.HCl (0.567 g, 3.5 mmol) were added to a solution of 4,4-difluoro-4-(diethylphosphonate)butanoic acid **18** (0.770 g, 2.9 mmol) in DCM (60 mL). The mixture was stirred overnight at room temperature. The reaction was quenched with a saturated aqueous solution of NH_4_Cl (60 mL), and the resulting mixture was extracted with DCM (3 × 20 mL). The organic layers were combined, washed with brine (60 mL), dried over anhydrous Na_2_SO_4_ and evaporated to dryness under reduced pressure. The product was purified by flash chromatography (EtOAc/PE 7:3) and obtained as a colorless oil (995 mg, 92% yield) as a mixture of two conformers in a 7:3 ratio respectively. R**_f_** = 0.35 (EtOAc/PE 7:3); ^1^H NMR (300 MHz, CDCl_3_): δ = 1.37 (6H, t, *J* = 7.1 Hz), 2.27–2.55 (7/10 of 4H, m), 2.59–2.76 (3/10 of 4H, m), 4.23–4.31 (4H, pseudo p, *J*_HH_ = *J*_PH_ = 7.2 Hz), 4.90 (2H, s), 7.35–7.39 (5H, m), 8.03 (3/10 of 1H, br s, NH), 8.73 (7/10 of 1H, br s, NH); ^13^C NMR (125 MHz, CDCl_3_): δ = 16.4 (CH_3_ × 2, d, *J*_CP_ = 5.5 Hz), 23.3–23.7 (CH_2_CF_2_*, m), 24.7–25.3 (CH_2_CF_2_, m), 28.0–28.7 (CH_2_CO*, m), 29.7 (CH_2_CO, dt, *J*_CF_ = 19.5 Hz, *J*_CP_ = 14.4 Hz), 64.3–65.0 (CH_2_ × 2, m), 78.2 (CH_2_Ph), 79.4 (CH_2_Ph*), 120.3 (CF_2_, dt, *J*_CF_ = 260.3 Hz, *J*_CP_ = 214.6 Hz), 128.6 (CH_ar_ × 2), 128.7 (CH_ar_), 129.1 (CH_ar_ × 2), 135.3 (C_ar_), 168.9 (CO), 175.1 (CO*); ^19^F NMR (282 MHz, CDCl_3_): δ = −112.52 (d, *J*_FP_ = 109.0 Hz), −111.34 (d, *J*_FP_ = 107.4 Hz); ^31^P NMR (121 MHz, CDCl_3_): δ = 6.37 (t, *J*_PF_ = 107.1 Hz), 6.65 (t, *J*_PF_ = 106.5 Hz.

*Diethyl 4-(N,N-benzyloxy-methyl)-amino-1,1-difluoro-4-oxobutyl phosphonate* (**19b**). To a stirred solution of diethyl 4-(*N*-benzyloxy)-amino-1,1-difluoro-4-oxobutyl phosphonate **18** (167 mg, 0.5 mmol) in anhydrous acetone (18 mL) was added anhydrous K_2_CO_3_ (82 mg, 0.6 mmol). The reaction was stirred under reflux for 30 min. Then, MeI (324 mg, 2.3 mmol) was added at room temperature and the solution was stirred overnight under reflux. The solution was filtered and evaporated to dryness under reduced pressure. The product was chromatographed on silica gel with gradient elution by EtOAc/PE 7:3 to 100% EtOAc to give **19b** as a yellow oil (165 mg, 94% yield) and as a single conformer. R_f_ = 0.55 (EtOAc); ^1^H NMR (500 MHz, CDCl_3_): δ = 1.36 (6H, t, J = 7.1 Hz), 2.32–2.44 (2H, m), 2.67–2.70 (2H, m), 3.20 (3H, s), 4.23–4.31 (4H, m), 4.84 (2H, s), 7.36–7.40 (5H, m); ^13^C NMR (125 MHz, CDCl_3_): δ = 16.4 (CH_3_ × 2, d, J_CP_ = 5.5 Hz), 24.0 (CH_2_CO), 28.9 (CH_2_CF_2_, dt, *J*_CF_ = 20.9 Hz, J_CP_ = 15.0 Hz), 33.75 (CH_3_N), 64.5 (CH_2_ × 2, d, J_CP_ = 6.7 Hz), 76.4 (CH_2_Ph), 120.4 (CF_2_, dt, *J*_CF_ = 257.7 Hz, J_CP_ = 215.2 Hz), 128.8 (CH_ar_ × 2), 129.1 (CH_ar_), 129.4 (CH_ar_ × 2), 134.2 (C_ar_), 173.1 (CO); ^19^F NMR (282 MHz, CDCl_3_): δ = −113.39 (d, J_FP_ = 108.1 Hz); ^31^P NMR (121 MHz, CDCl_3_): δ = 7.07 (t, J_PF_ = 107.7 Hz); HRMS (ESI^+^) *m*/*z* calcd for C_16_H_25_F_2_NO_5_P [M + H]^+^ 380.1433, found 380.1445.

*Diethyl 4-(N-hydroxyl)-amino-1,1-difluoro-4-oxobutyl phosphonate* (**20a**) [21]. To a stirred solution of diethyl 4-(*N*-benzyloxy)-amino-1,1-difluoro-4-oxobutyl phosphonate **19a** (217 mg, 0.6 mmol) in MeOH (15 mL) was added 10% *w*/*w* Pd on activated carbon (22 mg, 0.02 mmol). The reaction flask was then connected to a balloon of H_2_ at atmospheric pressure. Every 20 min, a slight vacuum was applied to the reaction flask, which was then backfilled with H_2_. When all starting material was consumed, the reaction was filtered over a pad of celite and evaporated to dryness under reduced pressure. The product was obtained pure in a quantitative yield (165 mg) as a colorless oil and as a mixture of two conformers *Z* and *E* in a 55:45 ratio respectively. R_f_ = 0.23 (EtOAc); ^1^H NMR (500 MHz, CDCl_3_): δ = 1.37 (6H, t, J = 7.0 Hz), 2.28–2.88 (4H, m), 4.28 (4H, pseudo p, J_HH_ = J_PH_ = 7.1 Hz), 5.65 (45/100 of 1H, br s, OH), 5.78 (55/100 of 1H, br s, OH), 8.04 (45/100 of 1H, br s, NH), 9.30 (55/100 of 1H, br s, NH); ^13^C NMR (125 MHz, CDCl_3_): δ = 16.4 (CH_3_ × 2, d, J_CP_ = 5.5 Hz), 24.7–24.9 (CH_2_CF_2_*, m), 27.2 (CH_2_CF_2_, q, *J*_CF_ = 4.8 Hz), 29.7 (CH_2_CO, dt, *J*_CF_ = 21.3 Hz, J_CP_ = 16.2 Hz), 29.9 (CH_2_CO*, dt, *J*_CF_ = 21.3 Hz, J_CP_ = 16.2 Hz), 64.7 (CH_2_* × 2, d, J_CP_ = 6.9 Hz), 65.0 (CH_2_ × 2, d, J_CP_ = 7.0 Hz), 120.2 (CF_2_, *J*_CF_ = 257.5 Hz, J_CP_ = 216.0 Hz), 169.0 (CO*), 173.3 (CO); ^19^F NMR (282 MHz, CDCl_3_): δ = −111.22 (d, J_FP_ = 107.3 Hz), −112.69 (d, J_FP_ = 107.7 Hz); ^31^P NMR (121 MHz, CDCl_3_): δ = 6.31 (t, J_PF_ = 107.2 Hz), 6.52 (t, J_PF_ = 107.7 Hz).

*Diethyl 4-(N,N-hydroxyl-methyl)-amino-1,1-difluoro-4-oxobutyl phosphonate* (**20b**). To a stirred solution of diethyl 4-(*N*,*N*-benzyloxy-methyl)-amino-1,1-difluoro-4-oxobutyl phosphonate **19b** (120 mg, 0.3 mmol) in MeOH (8 mL) and water (2.4 mL) was added 10% w/w Pd on activated carbon (12 mg, 0.01 mmol). The reaction flask was then connected to a balloon of H_2_ (1 atm). Every 20 min, a slight vacuum was applied to the reaction flask, which was then backfilled with H_2_. When all starting material was consumed, the reaction was filtered over celite and evaporated to dryness under reduced pressure. The pure product was obtained as a light yellow oil (90 mg, quantitative yield) and as a mixture of conformers in a 6:4 ratio. R_f_ = 0.33 (EtOAc); ^1^H NMR (500 MHz, CDCl_3_): δ = 1.38 (6H, t, J = 7.0 Hz), 2.29–2.51 (2H, m), 2.57–2.70 (4/10 of 2H, m), 2.70–2.86 (6/10 of 2H, m), 3.25 (6/10 of 3H, br s), 3.36 (4/10 of 3H, br s), 4.19–4.35 (4H, pseudo p, J_HH_ = J_PH_ = 7.3 Hz); ^13^C NMR (125 MHz, CDCl_3_): δ = 16.4 (CH_3_ × 2, d, J_CP_ = 4.7 Hz), 22.8 (CH_2_CO*), 24.0 (CH_2_CO), 27.8–28.1 (CH_2_CF_2_*, m), 29.0–30.3 (CH_2_CF_2_, m), 35.7 (CH_3_N*), 36.1 (CH_3_N), 64.7 (CH_2_ × 2, d, J_CP_ = 6.7 Hz), 65.0 (CH_2_* × 2, d, J_CP_ = 6.7 Hz), 119.9 (CF_2_*, dt, *J*_CF_ = 261.0 Hz, J_CP_ = 220.3 Hz), 120.3 (CF_2_, dt, *J*_CF_ = 261.0 Hz, J_CP_ = 220.3 Hz), 165.2 (CO*), 171.5 (CO*), 172.2 (CO), 173.6 (CO*); ^19^F NMR (282 MHz, CDCl_3_): δ = −110.9 (d, J_FP_ = 109.3 Hz), −112.4 (d, J_FP_ = 108.0 Hz), −113.4 (d, J_FP_ = 105.9 Hz), −113.6 (d, J_FP_ = 107.5 Hz); ^31^P NMR (121 MHz, CDCl_3_): δ = 6.61 (t, J_PF_ = 108.3 Hz), 6.75 (t, J_PF_ = 107.7 Hz); HRMS (ESI^+^) *m*/*z* calcd for C_9_H_18_F_2_NNaO_5_P [M + Na]^+^ 312.0783, found 312.0794.

*(1,1-Difluoro-4-(hydroxyamino)-4-oxobutyl)phosphonic acid* (**10a**). A solution of diethyl 4-(*N*-hydroxyl)-amino-1,1-difluoro-4-oxobutyl phosphonate **20a** (25 mg, 90.8 µmol) dissolved in DCM (0.34 mL) was cooled to 0 °C. TMSBr (0.12 mL, 0.9 mmol) was added dropwise at 0 °C then the reaction mixture was stirred overnight in the dark at room temperature. DCM and excess of TMSBr were evaporated under reduced pressure and the intermediate orange oil was treated with water (0.5 mL, 27.8 mmol) for 1.5 h. Removal of water under vacuum affords **10a** as a light orange solid (20 mg, quantitative yield) and as a mixture of conformers. R_f_ = 0.4 (EtOAc); ^1^H NMR (400 MHz, CD_3_OD): δ = 2.33–2.45 (2H, m), 2.50–2.53 (4/10 of 2H, m), 2.60 (6/10 of 2H, t, J = 7.5 Hz); ^13^C NMR (125 MHz, CD_3_OD): δ = 26.9–27.1 (CH_2_CF_2_, m), 28.0–28.1 (CH_2_CF_2_*, m), 30.3 (CH_2_CO_,_ dt, *J*_CF_ = 21.5 Hz, J_CP_ = 15.8 Hz), 30.6–31.1 (CH_2_CO*, m), 121.5 (CF_2_, dt, *J*_CF_ = 257.8 Hz, J_CP_ = 210.8 Hz), 174.2 (CO), 177.2 (CO*); ^19^F NMR (282 MHz, CD_3_OD): δ = −116.6 (d, J_FP_ = 104.6 Hz), −116.8 (d, J_FP_ = 104.4 Hz), −116.8 (d, J_FP_ = 104.1 Hz); ^31^P NMR (162 MHz, CD_3_OD): δ = 5.12 (t, J_PF_ = 104.0 Hz), 5.21 (t, J_PF_ = 104.4 Hz), 5.32 (t, J_PF_ = 104.9 Hz); HRMS (ESI^+^) *m*/*z* calcd for C_4_H_9_F_2_NO_5_P [M + H]^+^ 220.0181, found 220.0192.

*(1,1-Difluoro-4-(hydroxy(methyl)amino)-4-oxobutyl)phosphonic acid* (**10b**). A solution of diethyl 4-(*N*,*N*-hydroxyl-methyl)-amino-1,1-difluoro-4-oxobutyl phosphonate **20b** (20 mg, 69.2 µmol) dissolved in DCM (0.26 mL) was cooled to 0 °C. TMSBr (0.10 mL, 0.7 mmol) was added dropwise at 0 °C then the reaction mixture was stirred overnight in the dark at room temperature. DCM and excess of TMSBr were evaporated under reduced pressure and the intermediate orange oil was treated with water (0.5 mL, 27.8 mmol) for 1.5 h. Removal of water under vacuum affords **10b** as an orange oil (16 mg, quant. yield) and as a mixture of conformers. R_f_ = 0.1 (EtOAc); ^1^H NMR (400 MHz, CD_3_OD): δ = 2.28–2.47 (2H, m), 2.56–2.63 (4/10 of 2H, m), 2.74–2.78 (6/10 of 2H, m), 2.95 (6/10 of 3H, s), 3.21 (4/10 of 3H, s); ^13^C NMR (125 MHz, CD_3_OD): δ = 25.0–25.2 (CH_2_CF_2_*, m), 26.8–27.0 (CH_2_CF_2_, m), 30.2 (CH_2_CO*, dt, *J*_CF_ = 19.9 Hz, J_CP_ = 15.7 Hz), 30.3 (CH_2_CO, dt, *J*_CF_ = 21.2 Hz, J_CP_ = 15.7 Hz), 36.4 (CH_3_N*), 38.1 (CH_3_N), 121.7 (CF_2_, td, *J*_CF_ = 258.4 Hz, J_CP_ = 212.1 Hz), 174.0 (CO*), 174.2 (CO); ^19^F NMR (282 MHz, CD_3_OD): δ = −118.1 (d, J_FP_ = 104.1 Hz), −118.4 (d, J_FP_ = 104.1 Hz); ^31^P NMR (162MHz, CD_3_OD: δ = 5.21 (t, J_PF_ = 103.7 Hz), 5.33 (t, J_PF_ = 105.3 Hz), 5.48 (t, J_PF_ = 105.8 Hz); HRMS (ESI^−^) *m*/*z* calcd for C_5_H_10_F_2_NO_5_P [M − H]^−^ 232.0192, found 232.0206.

### 4.2. Biological Activity

#### 4.2.1. His-Tagged DXR Activity

The assays were performed at 37 °C in a 50 mM Tris/HCl buffer pH 7.5 containing 3 mM MgCl_2_ and 2 mM DTT. The concentrations of DXP and NADPH were 480 µM and 160 µM respectively. The decrease of absorbance at 340 nm due to NADPH oxidation was monitored to determine the initial rates. The retained values were the average of at least two measurements. The relative average deviation must be lower than 4%.

#### 4.2.2. Inhibition of His-Tagged DXR 

Fosmidomycin was purchased from Fujisawa Pharmaceutical. Inhibitor concentrations in the stock solutions were verified by spectrophotometric phosphorus determination [28]. The study compounds **9b** and **10a**, **10b** were tested against *E. coli* DXR using a photometric assay that was described earlier [8]. H-DXR was pre-incubated during 2 min in the presence of the inhibitors **9b**, **10a** and **10b** at different concentrations and NADPH. DXP was then added to measure the residual activity. The inhibitory potential of the tested compounds was quantified by determining the IC_50_ values. They were obtained by plotting the percentage of residual activity versus the Log of inhibitor concentration.

#### 4.2.3. Bacterial Growth Inhibition

The antimicrobial activity of hydroxamic acids **9b**, **10a** and **10b** against *E. coli* XL1 Blue [8] and fosmidomycin-resistant strain *E. coli* FosR [7], was determined using the paper disc diffusion method. LB agar plates (9 cm diameter) were inoculated with a suspension of bacteria (200 μL, mid-exponential phase). Paper discs (Durieux no. 268, diameter 6 mm) impregnated with a volume ≤8 μL of fosmidomycin derivatives were placed on Petri dishes. Growth inhibition was examined after 24 h incubation at 37 °C.

## Figures and Tables

**Table 1:** Inhibition of *E. coli* H6-DXR.

Compounds	IC_50_ (nM)
fosmidomycin, **3a**	42
FR-900098, **3b**	4
**4a**	180 **
**4b**	48 **
fosfoxacin, **5a**	342 *
**5b**	77 *
**6a**	2600 *
**6b**	46 *
**9a**	-
**9b**	9
**10a**	4600
**10b**	17

Mean from at least 2 different assays. Errors were <5%. * and ** values obtained from references [9] and [8] respectively.

**Table 2:** *E. coli* XL1 Blue growth inhibition on LB solid medium.

Compounds	nmoles/Disc	Growth Inhibition Zone (mm)
fosmidomycin, **3a**	2	35
FR-900098, **3b**	2 *	32 *
**4a**	400 **	12 **
**4b**	80 **	30 **
**6a**	400 *	<10 *
**6b**	160 *	20 *
**9b**	2	15
**10a**	3	-
**10b**	1.5	20

* and ** values obtained from references [9] and [8] respectively.

## Data Availability

The data presented in this study are contained within the article and are also available in the Supplementary Materials.

## Supplementary Materials

The following are available online.^1^H-NMR, ^13^C-NMR, ^19^F-NMR and ^31^P-NMR spectra of compounds **5**–**20** are included.

## Sample Availability

Not available.
