# Supplementary material for: α,α-Difluorophosphonohydroxamic Acid Derivatives among the Best Antibacterial Fosmidomycin Analogues
Source: Molecules. 2021 Aug 23;26(16):5111. doi: 10.3390/molecules26165111 (PMC8397956; doi:10.3390/molecules26165111)

α,α-Difluorophosphonohydroxamic acid derivatives among the best antibacterial fosmidomycin analogues

Aurore Dreneau, Fanny S. Krebs, Mathilde Munier, Chheng Ngov, Denis Tritsch, Didier Lièvremont, Michel Rohmer and Catherine Grosdemange-Billiard*

Laboratoire de Chimie et Biochimie de Molécules Bioactives – Université de Strasbourg/CNRS, UMR 7177, Institut Le Bel, 4 rue Blaise Pascal, 67081 Strasbourg, France

^{1}H, ^{13}C, ^{31}P, ^{19}F NMR spectra

Compound **12**	2
Compound **13**	4
Compound **14**	5
Compound **15a**	7
Compound **15b**	9
Compound **16a**	11
Compound **16b**	13
Compound **17**	15
Compound **18**	17
Compound **19a**	19
Compound **19b**	21
Compound **20a**	23
Compound **20b**	25
Compound **9b**	27
Compound **10a**	29
Compound **10b**	31

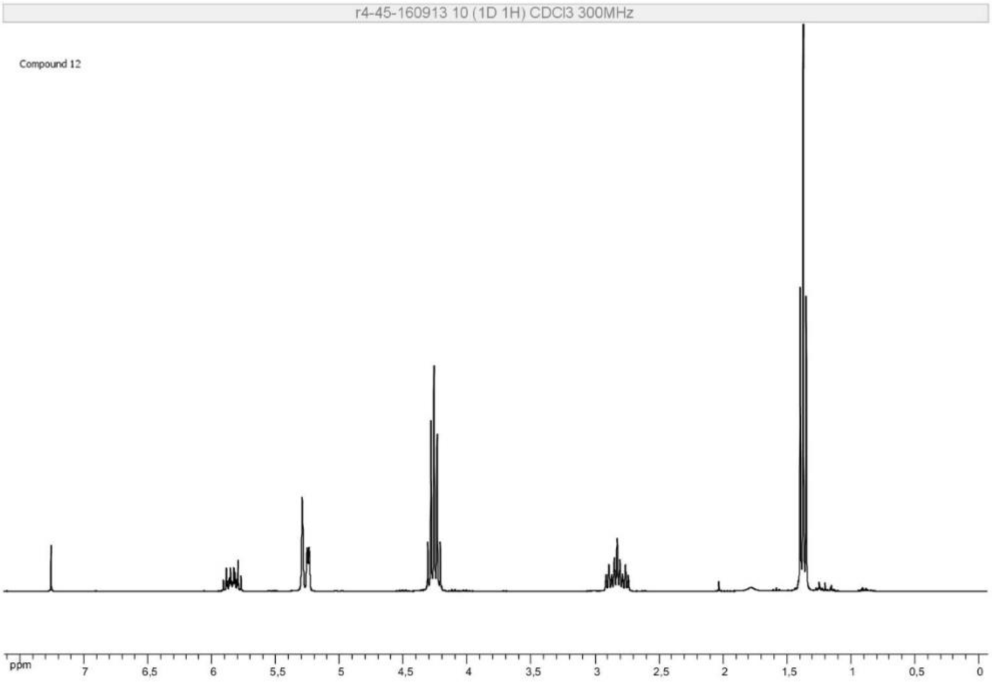

Compound **19b**

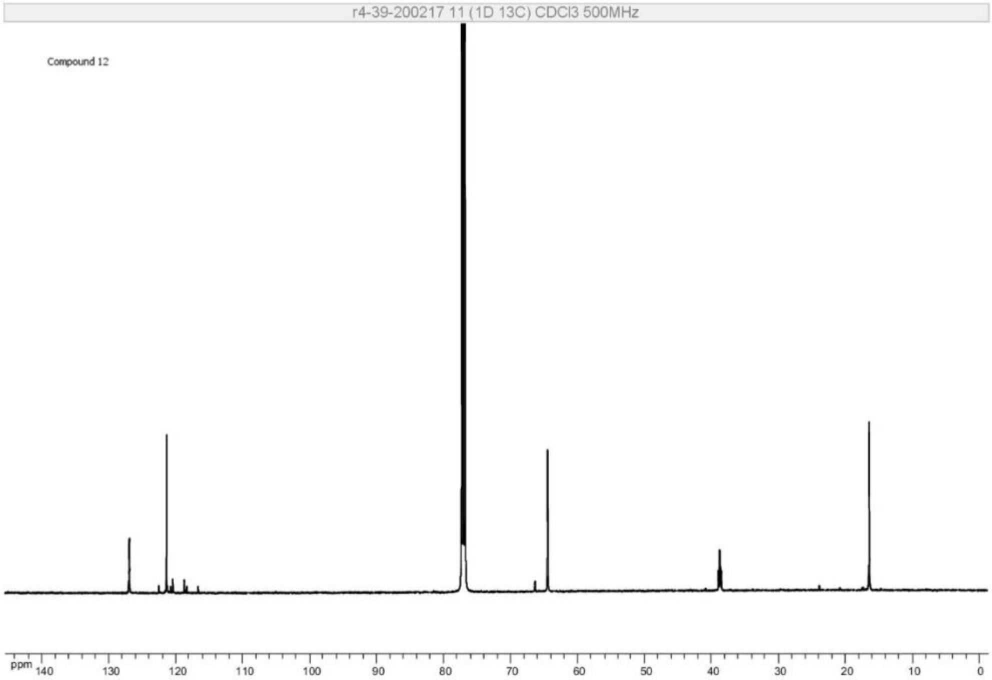

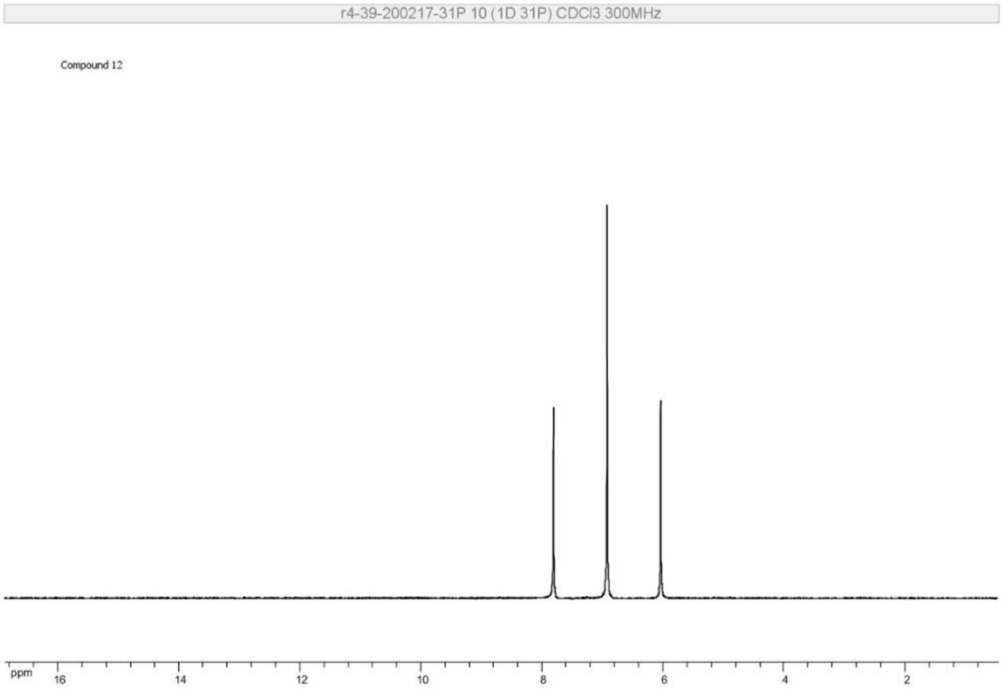
r4-39-200217-31P 10 (1D 31P) CDCl3 300MHz
Compound 12
ppm 16 14 12 10 8 6 4 2

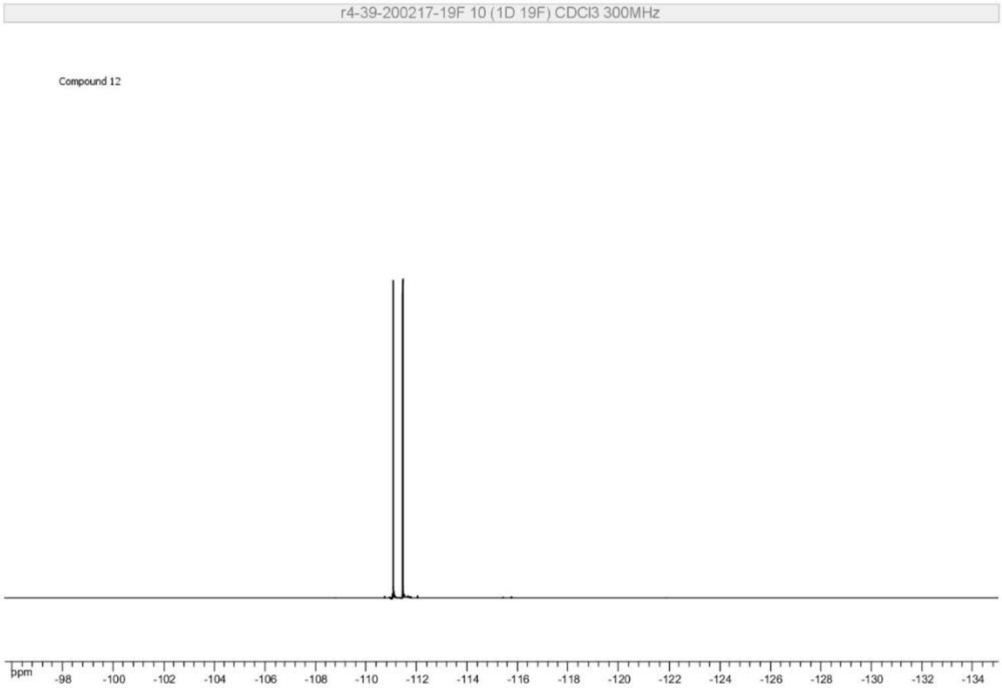
r4-39-200217-19F 10 (1D 19F) CDCl3 300MHz
Compound 12
ppm -98 -100 -102 -104 -106 -108 -110 -112 -114 -116 -118 -120 -122 -124 -126 -128 -130 -132 -134

Compound **13**

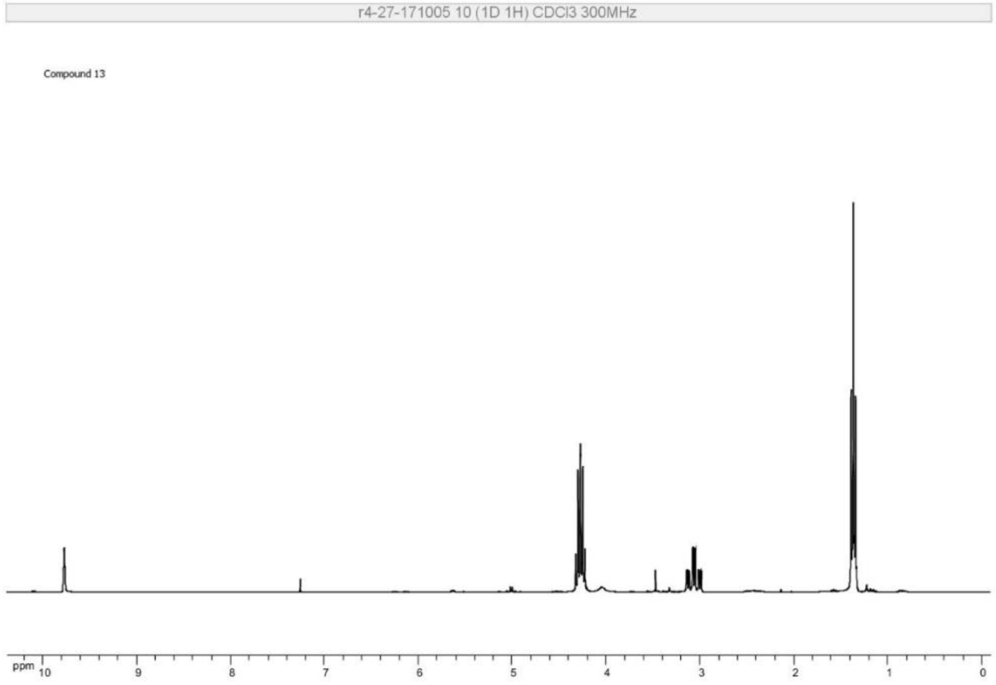
r4-27-171005 10 (1D 1H) CDCl3 300MHz
Compound 13
ppm
10
9
8
7
6
5
4
3
2
1
0

Compound **14**

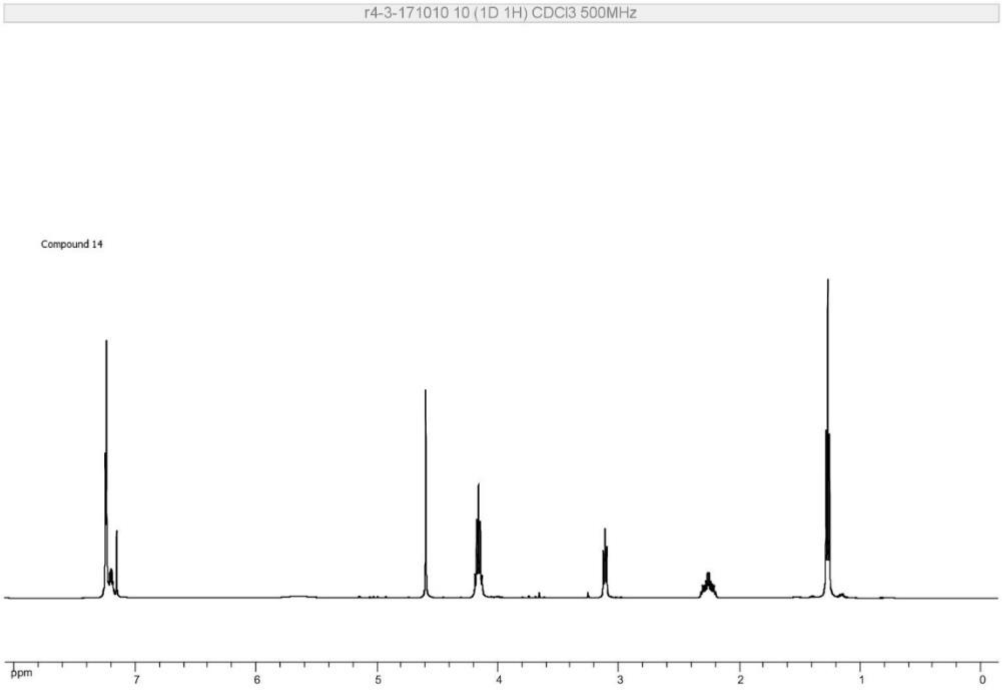
r4-3-171010 10 (1D 1H) CDCl3 500MHz
Compound 14
ppm
7
6
5
4
3
2
1
0

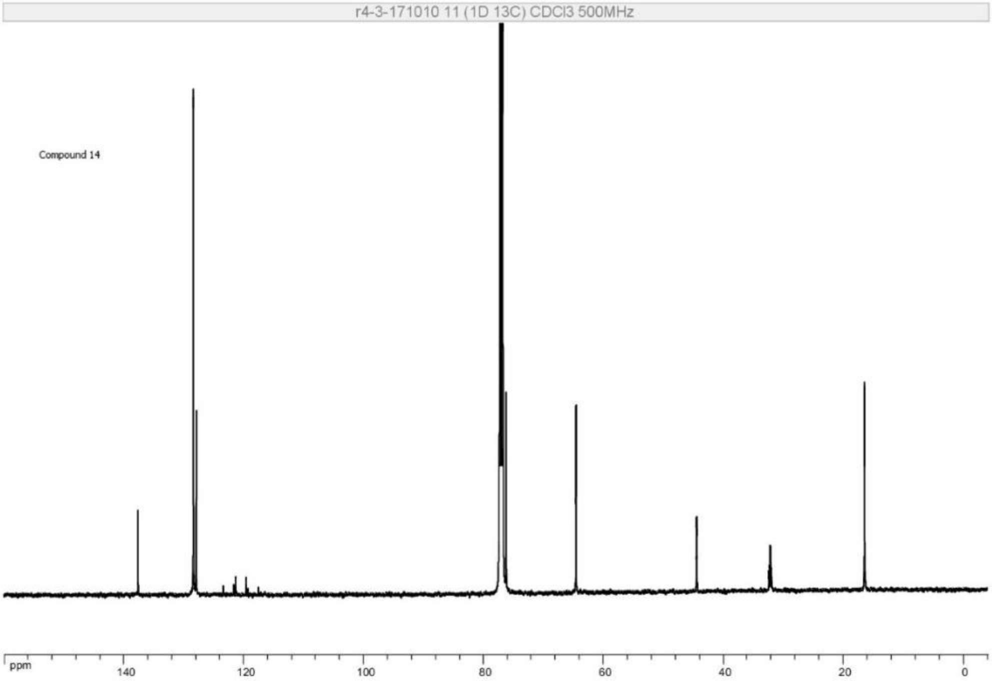
r4-3-171010 11 (1D 13C) CDCl3 500MHz
Compound 14
ppm
140
120
100
80
60
40
20
0

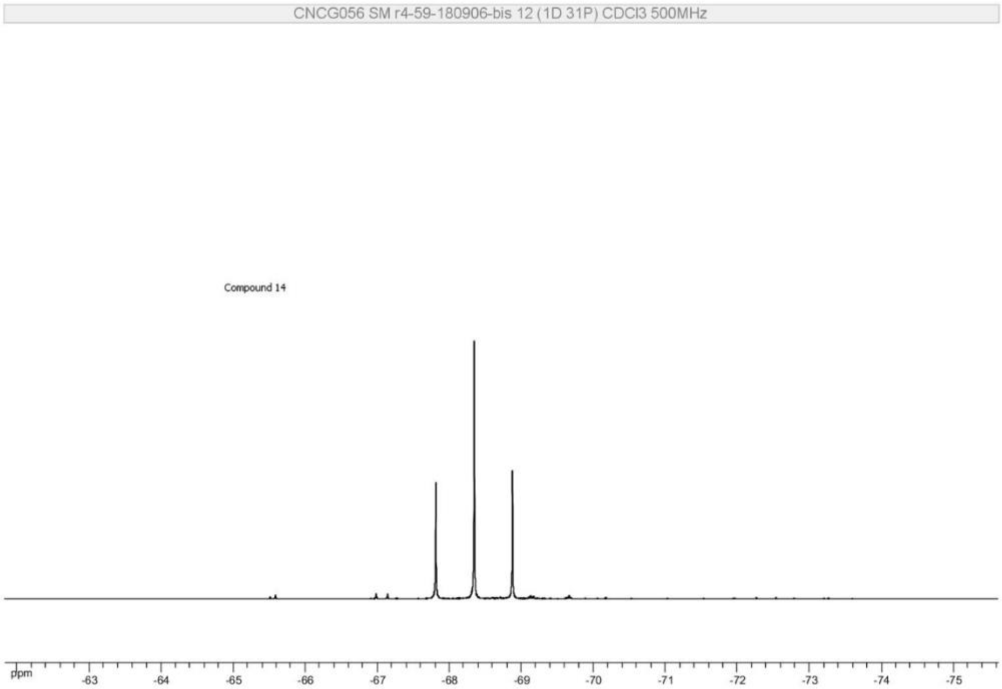
CNCG056 SM r4-59-180906-bis 12 (1D 31P) CDCl3 500MHz
Compound 14
ppm
-63
-64
-65
-66
-67
-68
-69
-70
-71
-72
-73
-74
-75

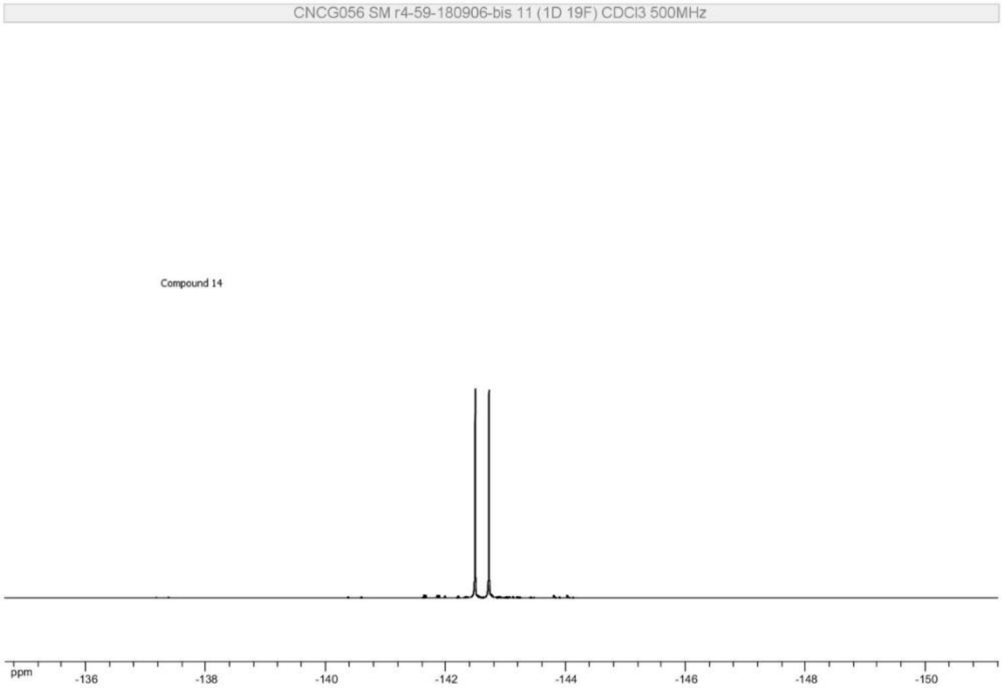
CNCG056 SM r4-59-180906-bis 11 (1D 19F) CDCl3 500MHz
Compound 14
ppm
-136
-138
-140
-142
-144
-146
-148
-150

Compound **15a**

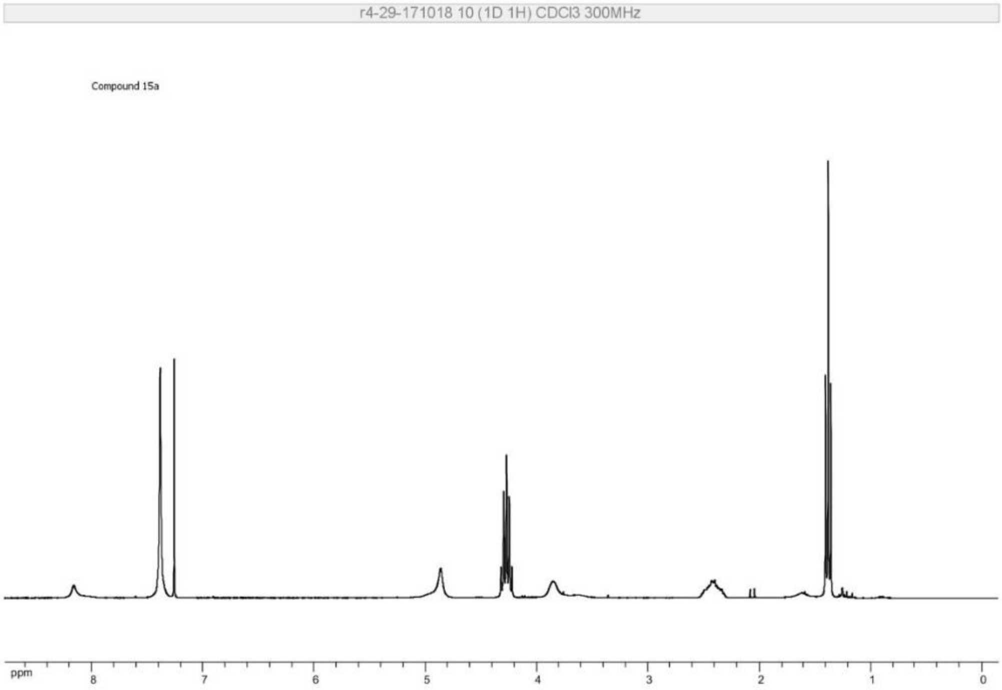

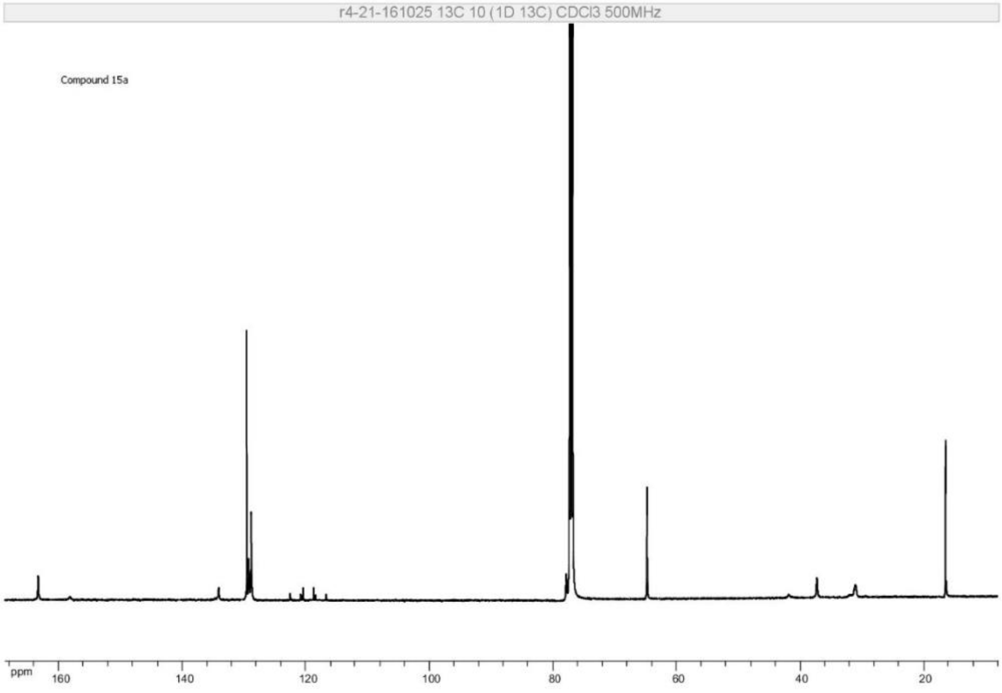

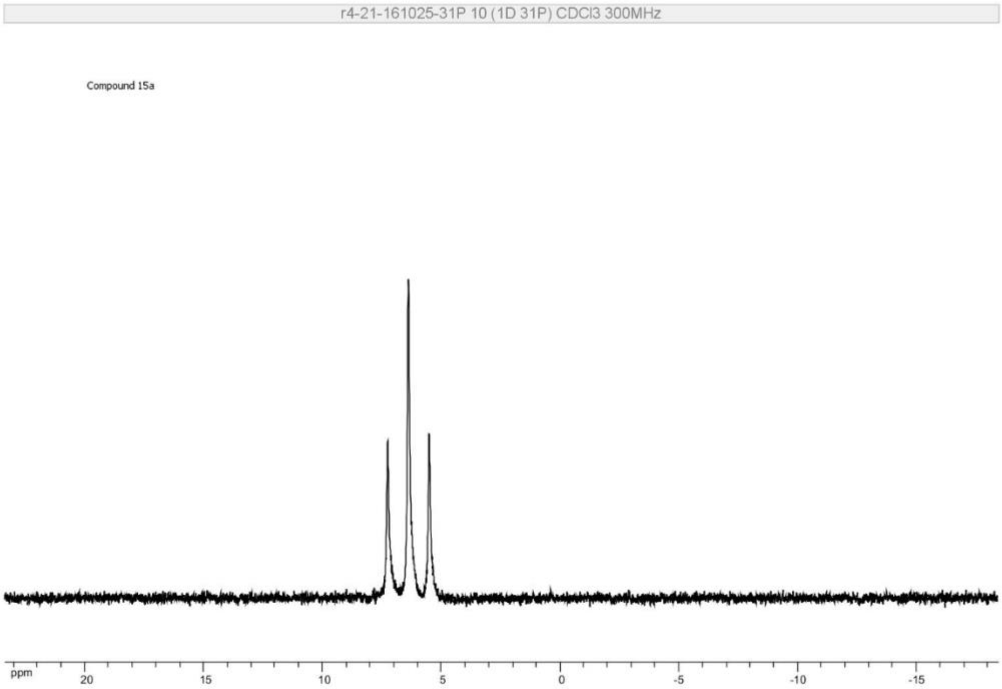
r4-21-161025-31P 10 (1D 31P) CDCl3 300MHz
Compound 15a
ppm
20
15
10
5
0
-5
-10
-15

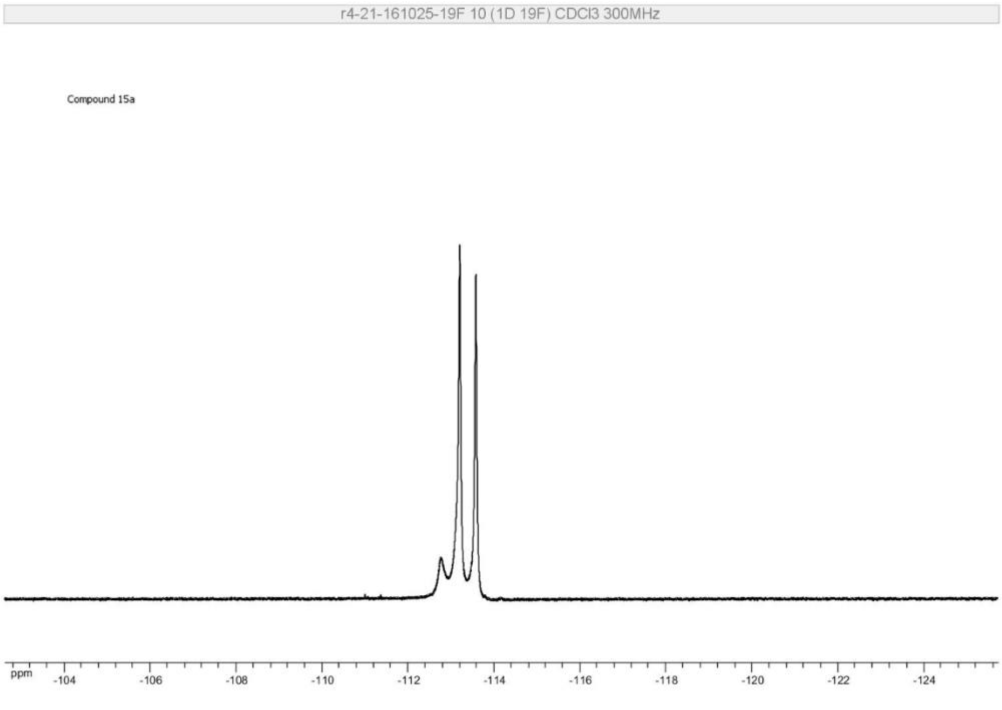
r4-21-161025-19F 10 (1D 19F) CDCl3 300MHz
Compound 15a
ppm
-104
-106
-108
-110
-112
-114
-116
-118
-120
-122
-124

Compound **15b**

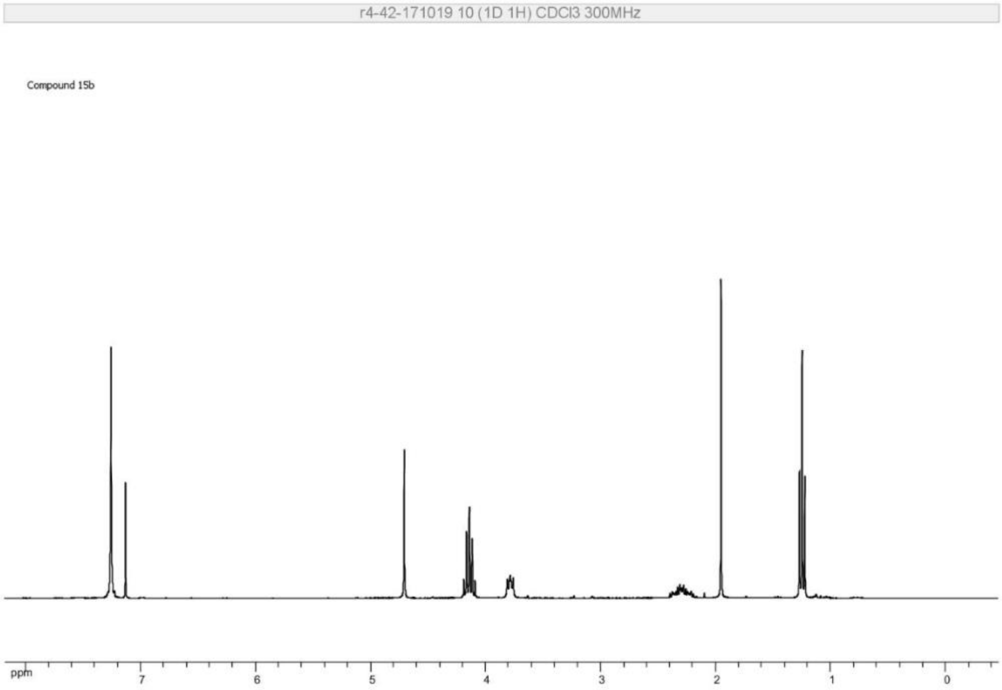

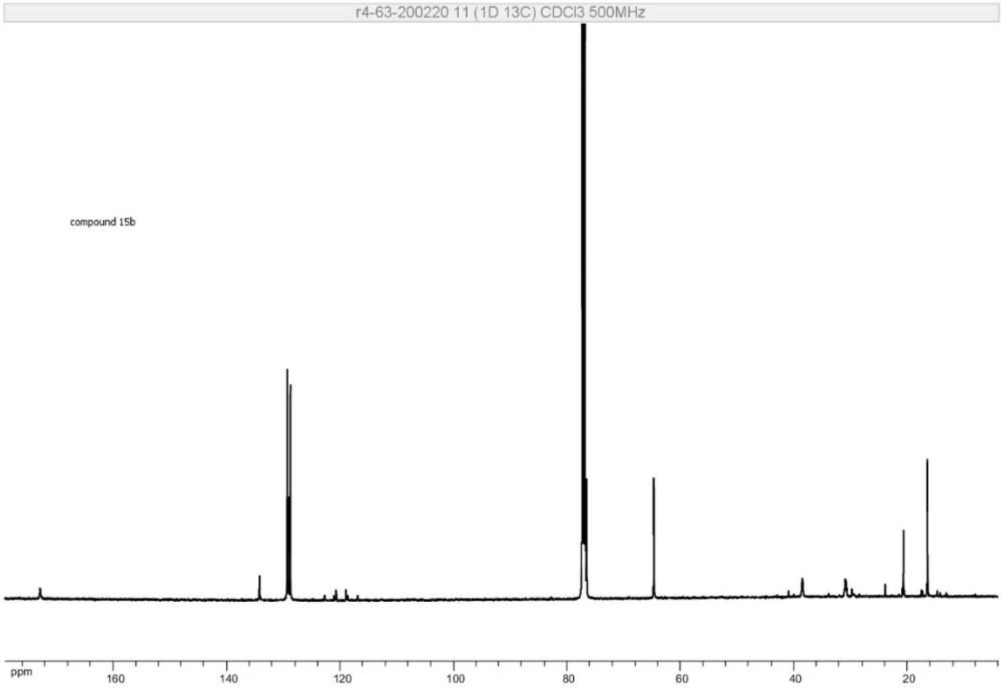

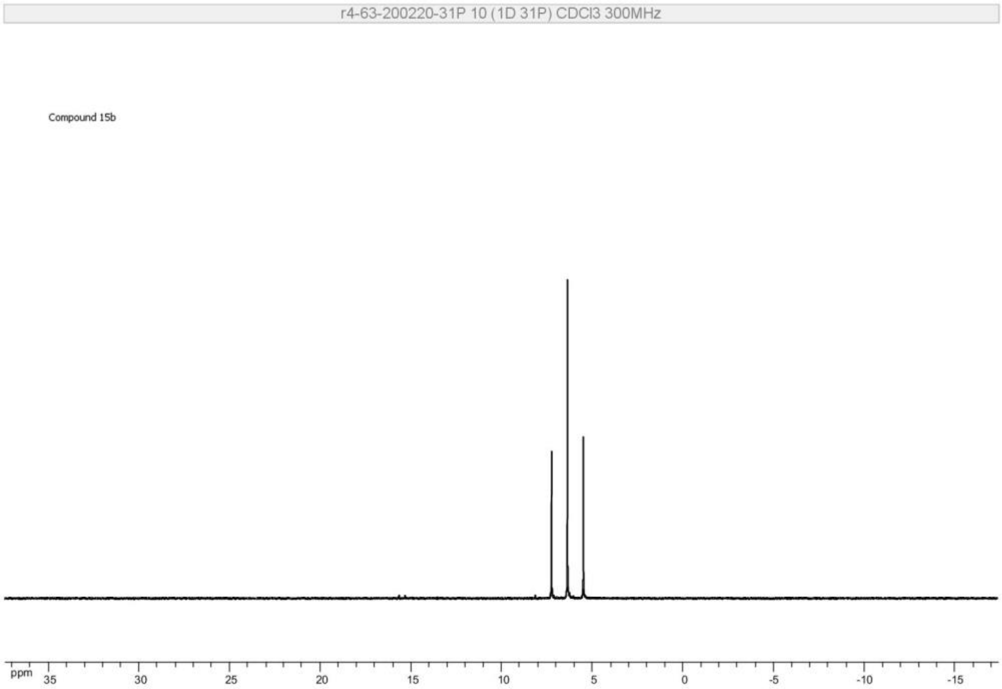
r4-63-200220-31P 10 (1D 31P) CDCl3 300MHz
Compound 15b
ppm 35 30 25 20 15 10 5 0 -5 -10 -15

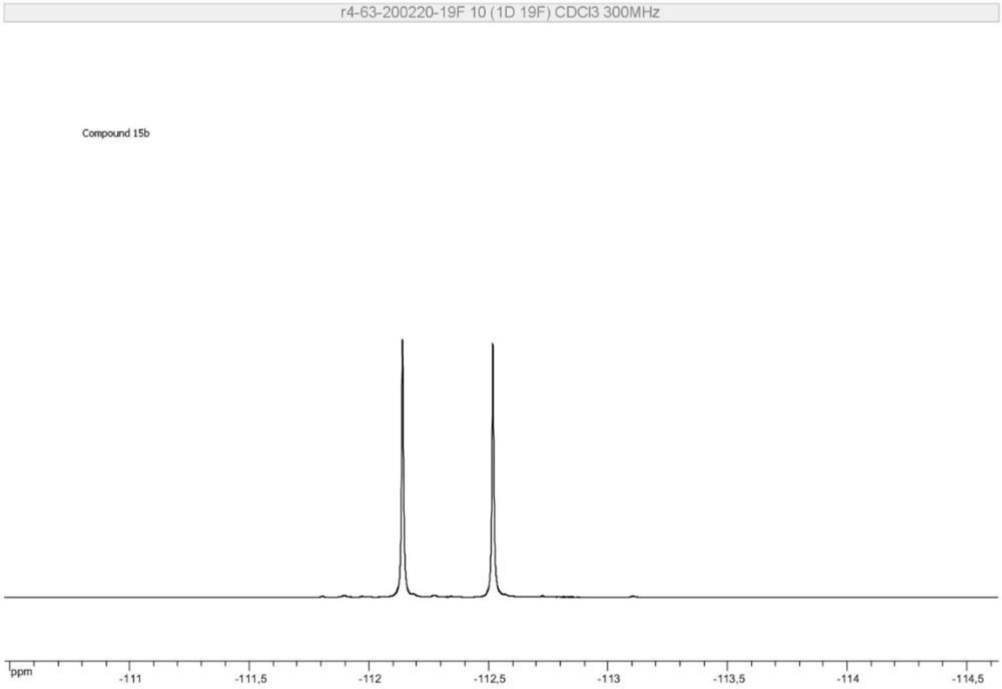
r4-63-200220-19F 10 (1D 19F) CDCl3 300MHz
Compound 15b
ppm -111 -111,5 -112 -112,5 -113 -113,5 -114 -114,5

Compound **16a**

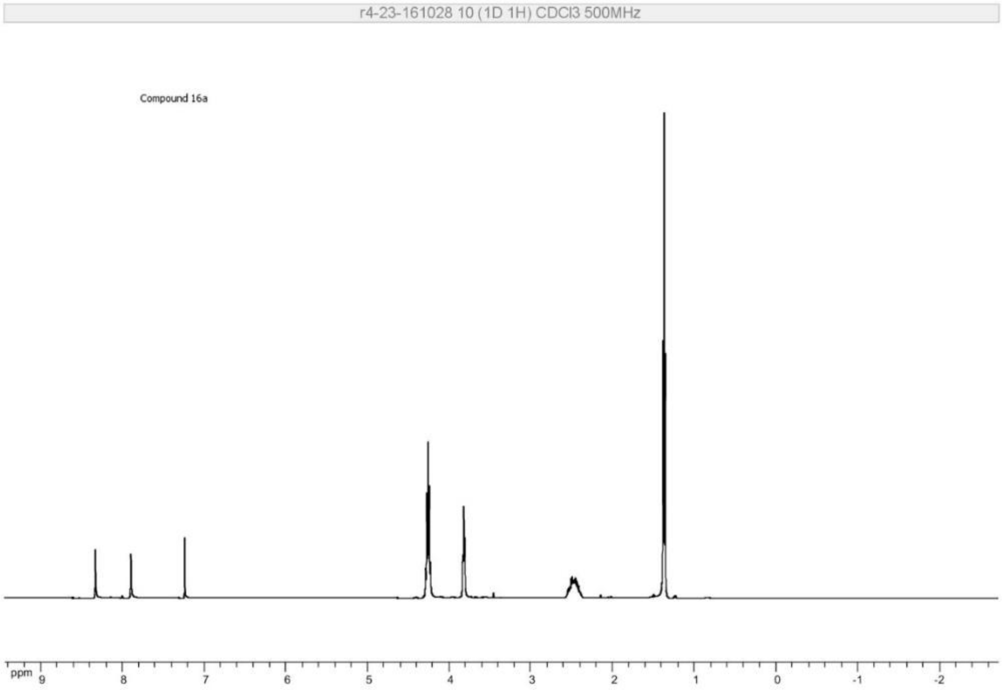

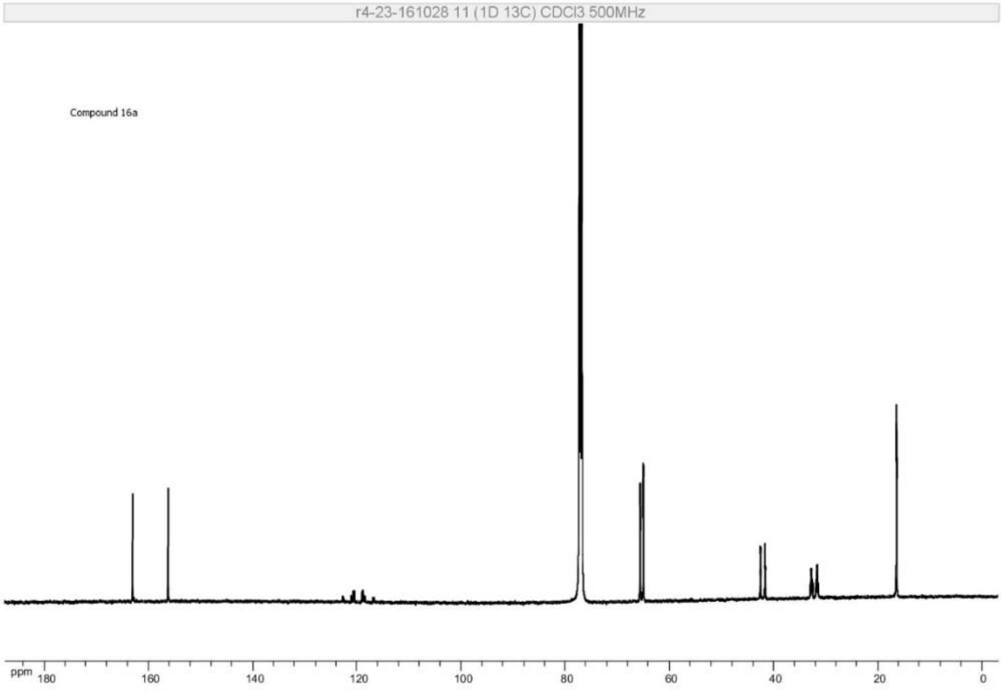

r4-23-161028-31P 10 (1D 31P) CDCl3 300MHz

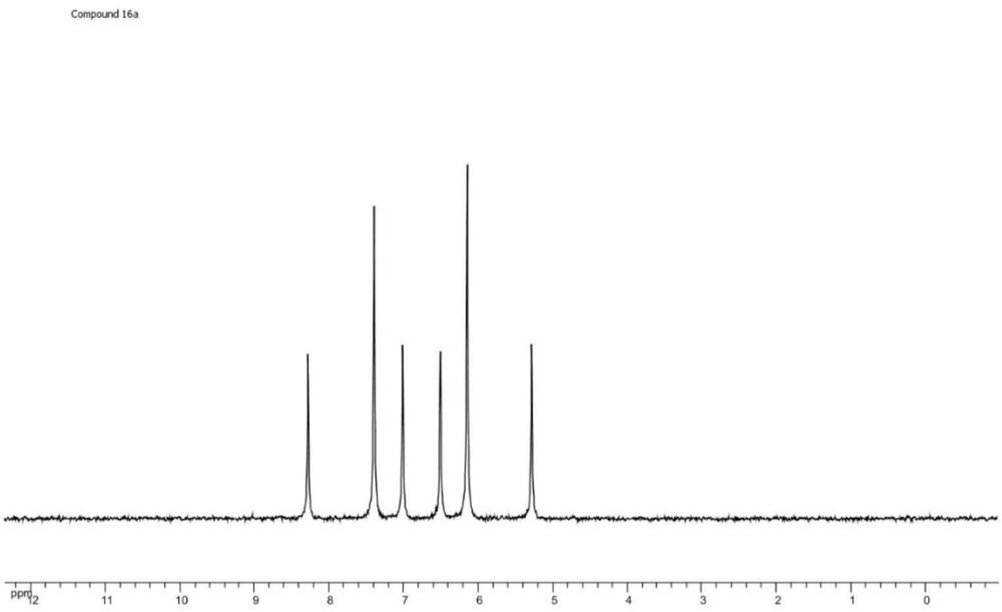

r4-23-161028-19F 10 (1D 19F) CDCl3 300MHz

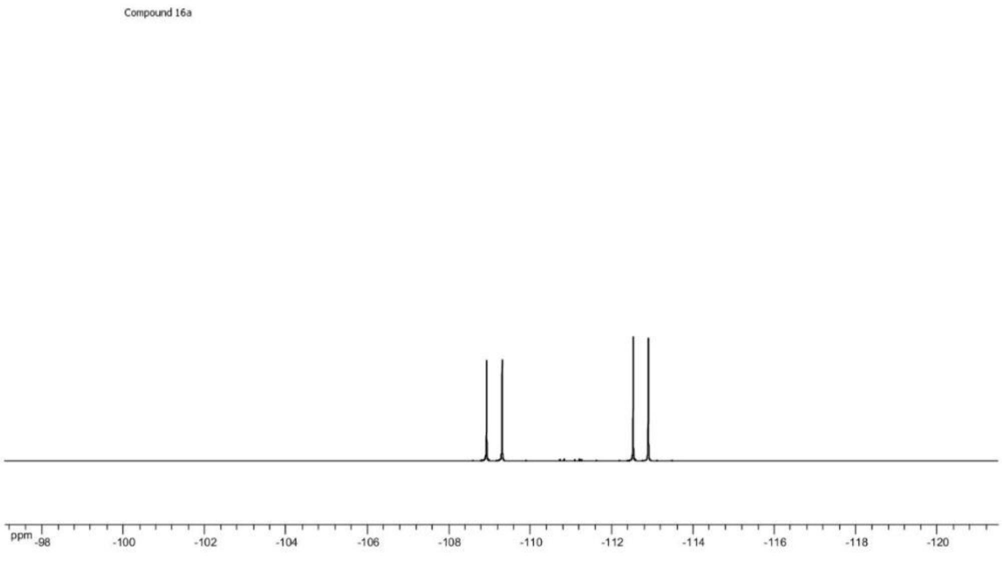

Compound **16b**

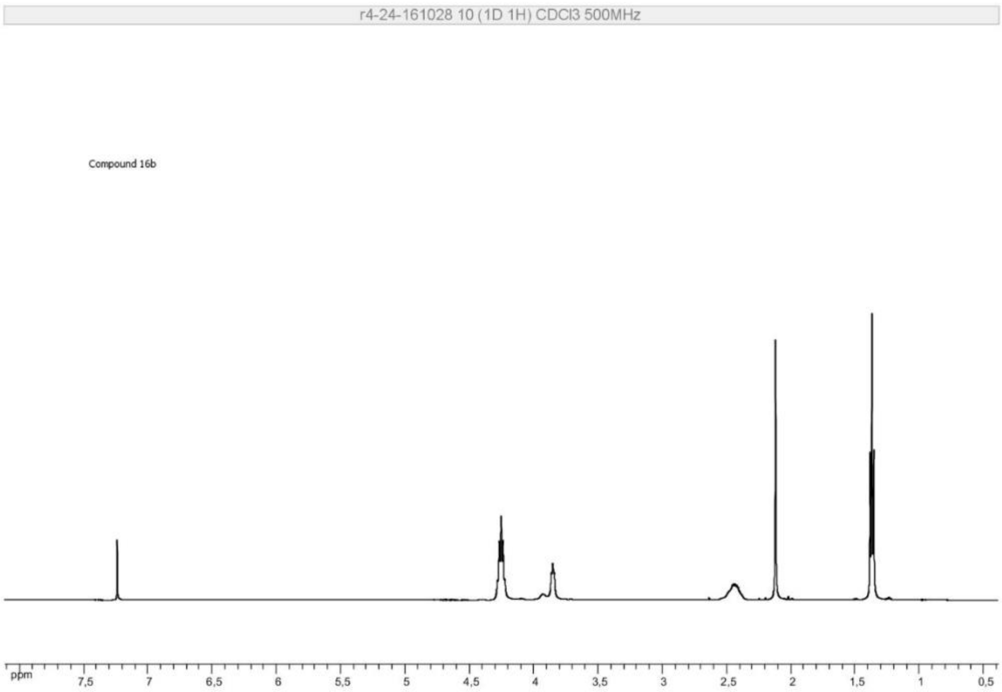

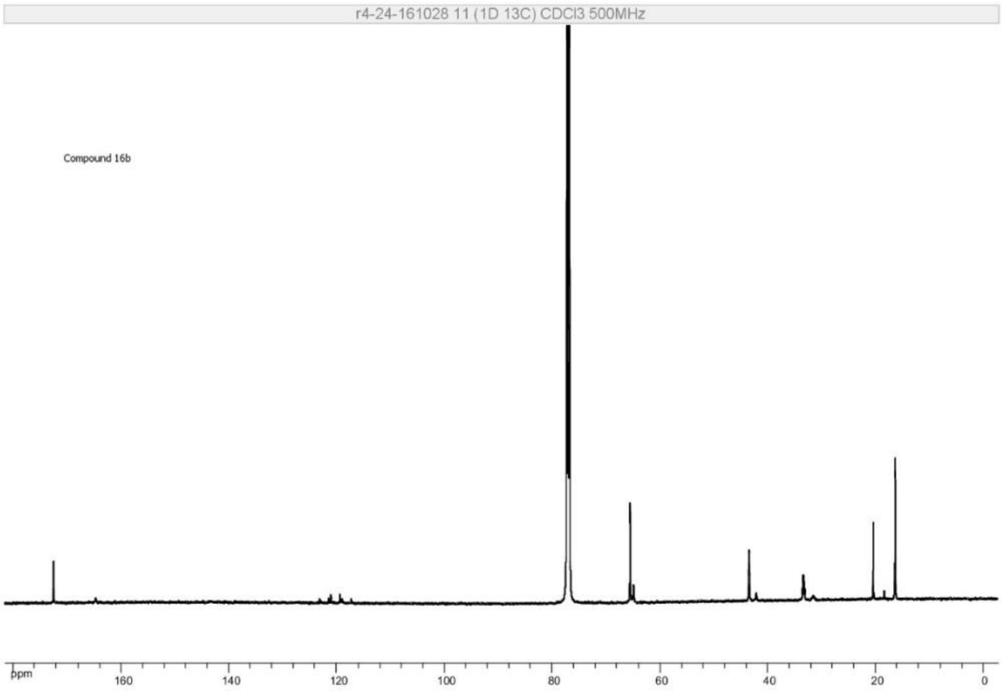

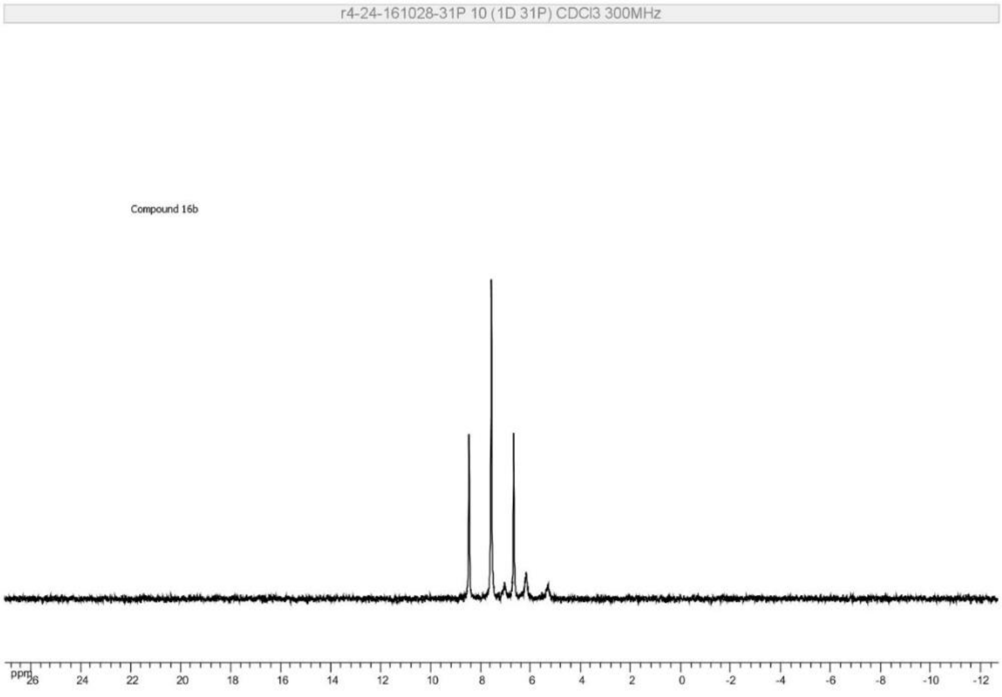
r4-24-161028-31P 10 (1D 31P) CDCl3 300MHz
Compound 16b
ppm 26 24 22 20 18 16 14 12 10 8 6 4 2 0 -2 -4 -6 -8 -10 -12

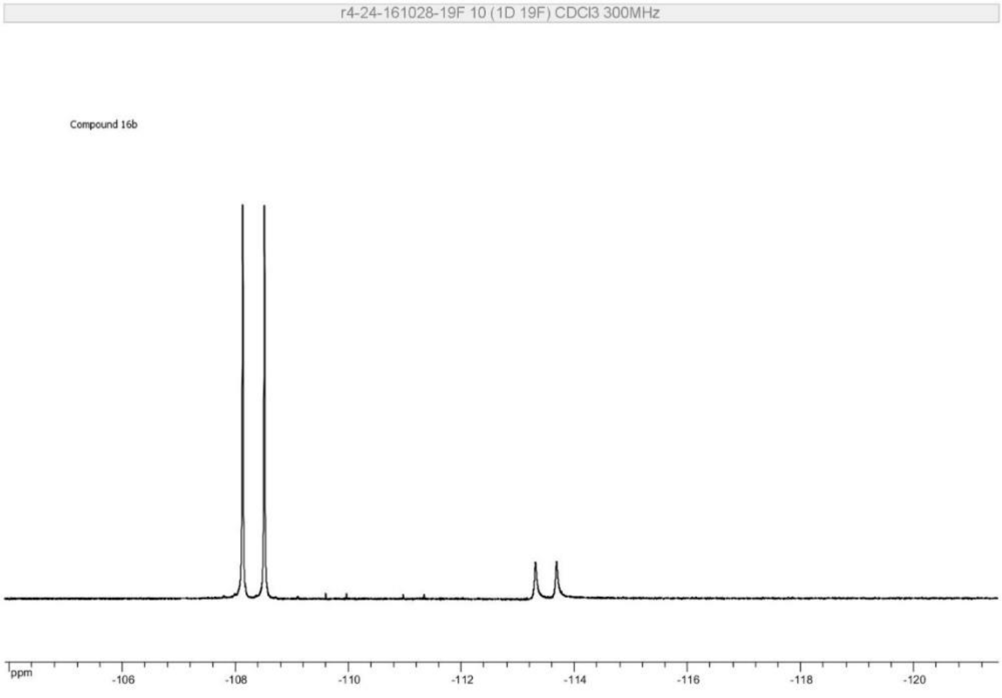
r4-24-161028-19F 10 (1D 19F) CDCl3 300MHz
Compound 16b
ppm -106 -108 -110 -112 -114 -116 -118 -120

Compound **17**

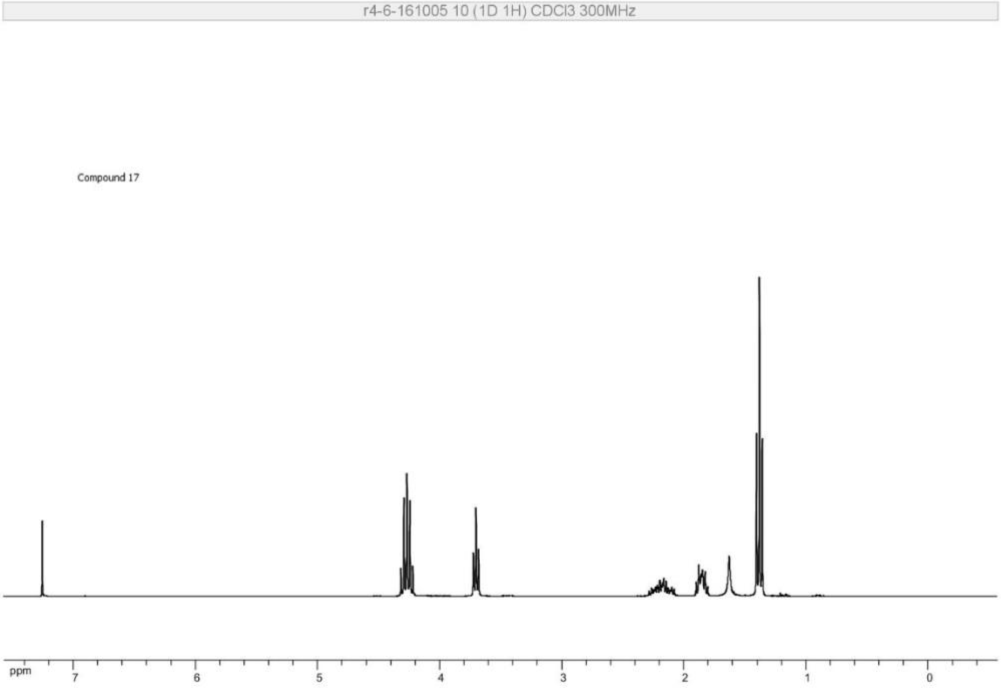
r4-6-161005 10 (1D 1H) CDCl3 300MHz
Compound 17
ppm
7
6
5
4
3
2
1
0

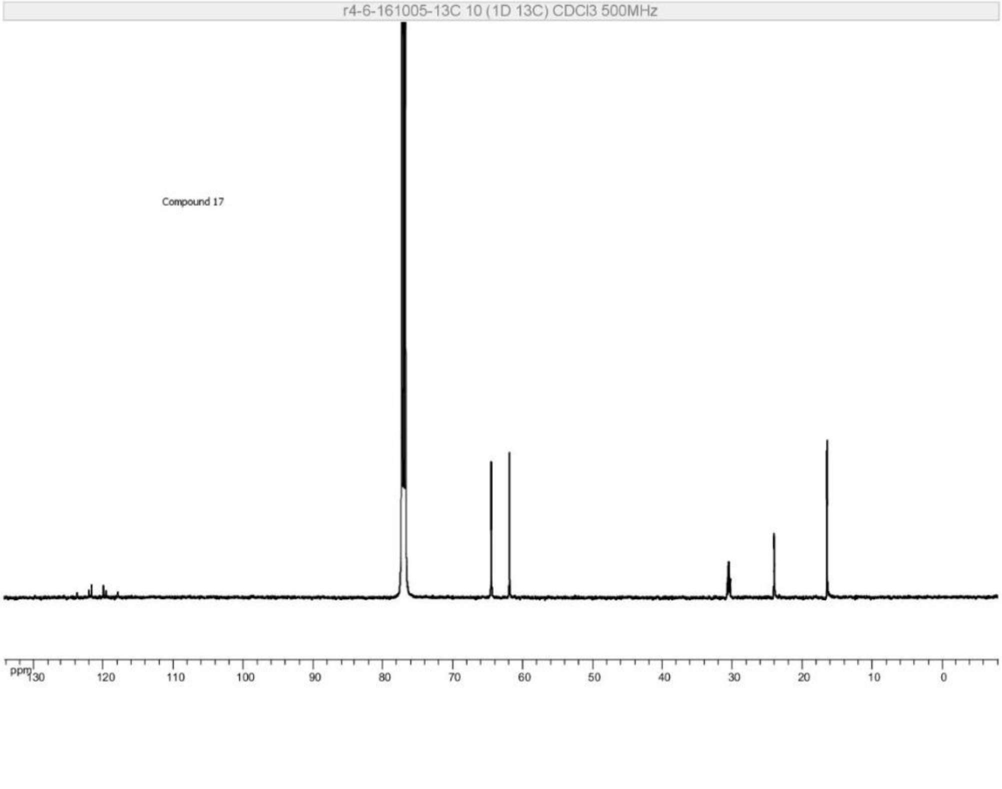
r4-6-161005-13C 10 (1D 13C) CDCl3 500MHz
Compound 17
ppm
130
120
110
100
90
80
70
60
50
40
30
20
10
0

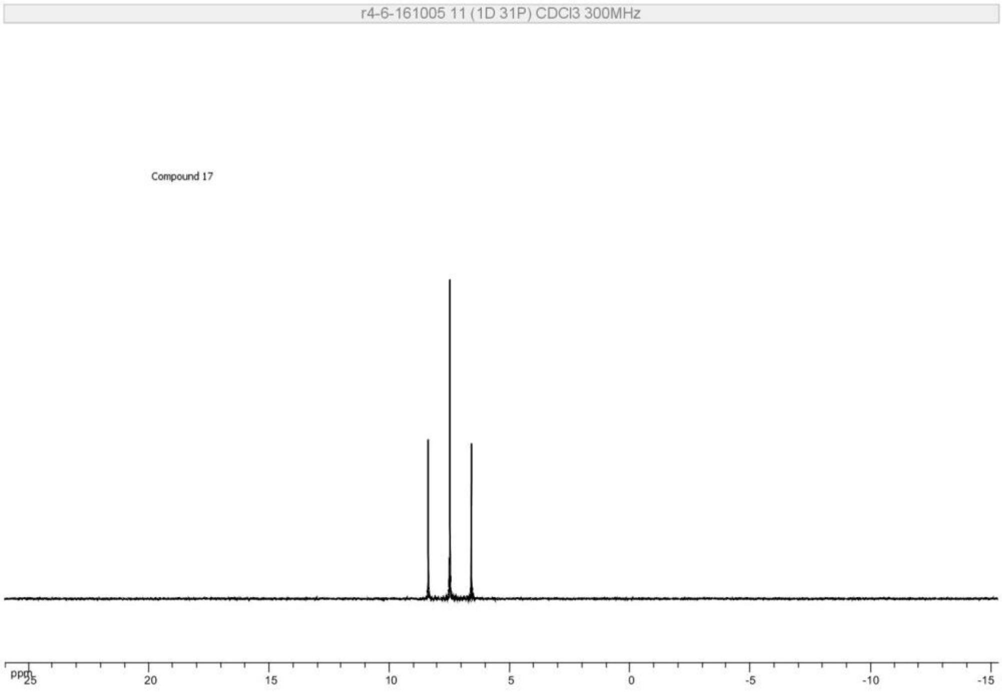
r4-6-161005 11 (1D 31P) CDCl3 300MHz
Compound 17
ppm
25
20
15
10
5
0
-5
-10
-15

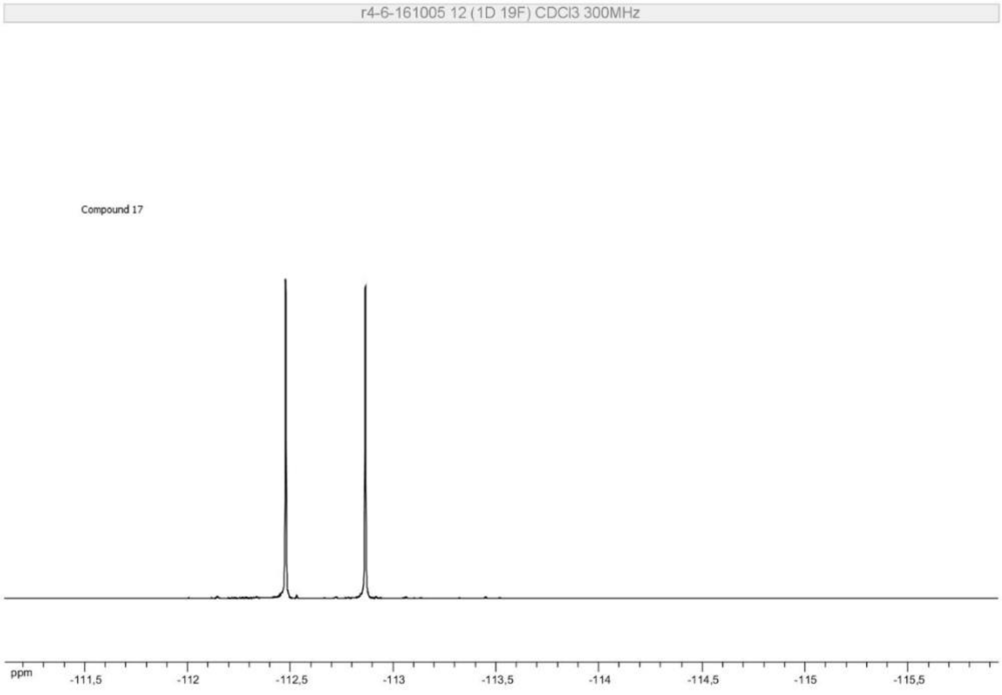
r4-6-161005 12 (1D 19F) CDCl3 300MHz
Compound 17
ppm
-111,5
-112
-112,5
-113
-113,5
-114
-114,5
-115
-115,5

Compound **18**

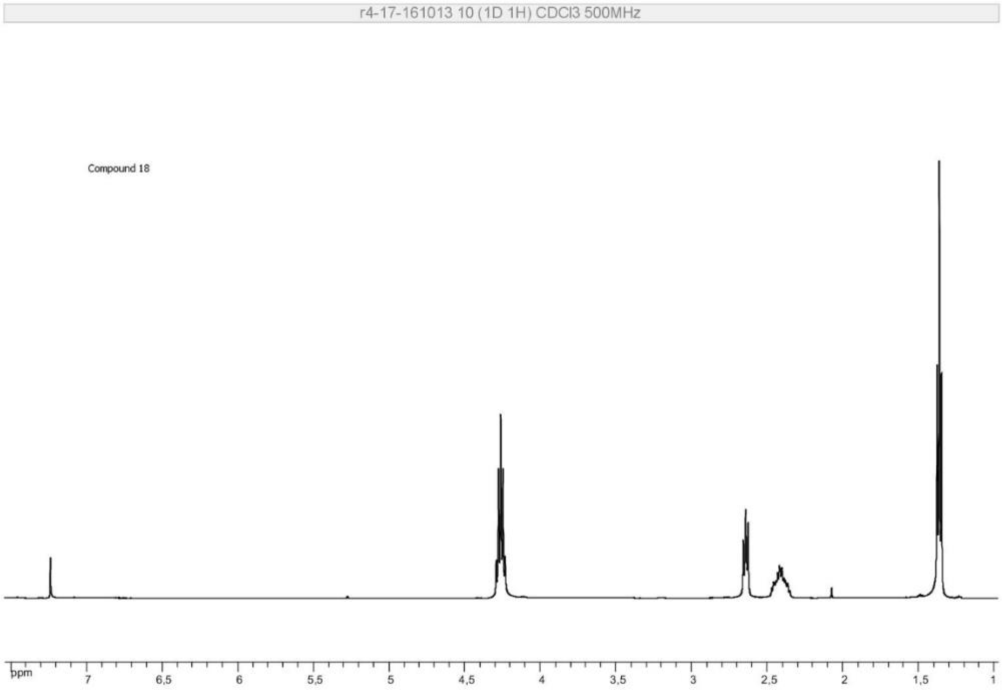

r4-17-161013 10 (1D 1H) CDCl3 500MHz
Compound 18
ppm
7
6,5
6
5,5
5
4,5
4
3,5
3
2,5
2
1,5
1

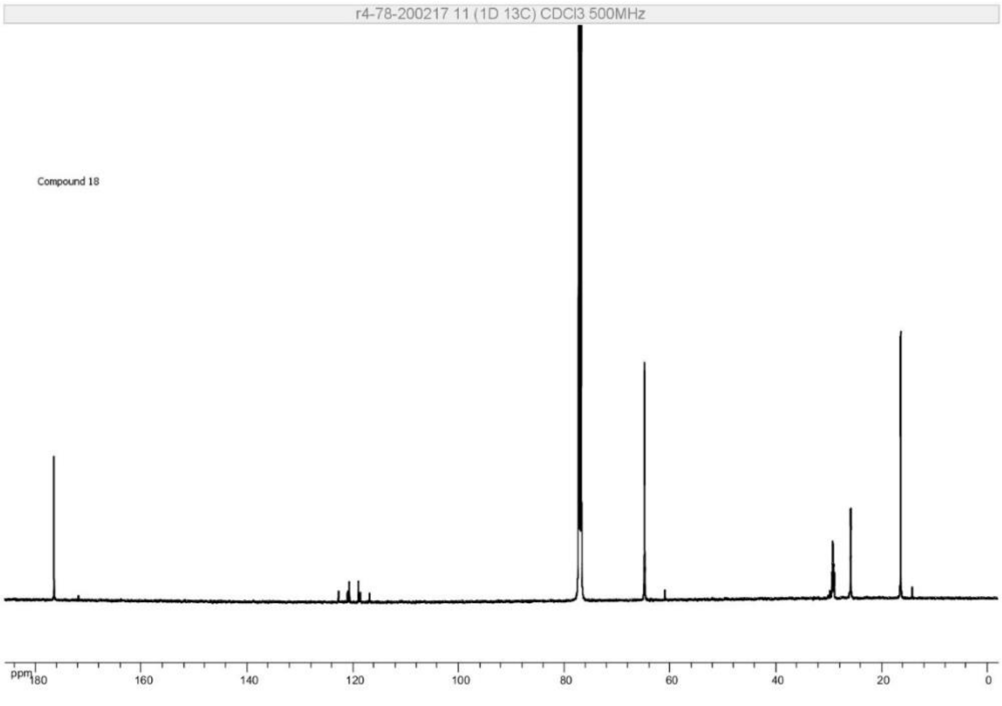

r4-78-200217 11 (1D 13C) CDCl3 500MHz
Compound 18
ppm
180
160
140
120
100
80
60
40
20
0

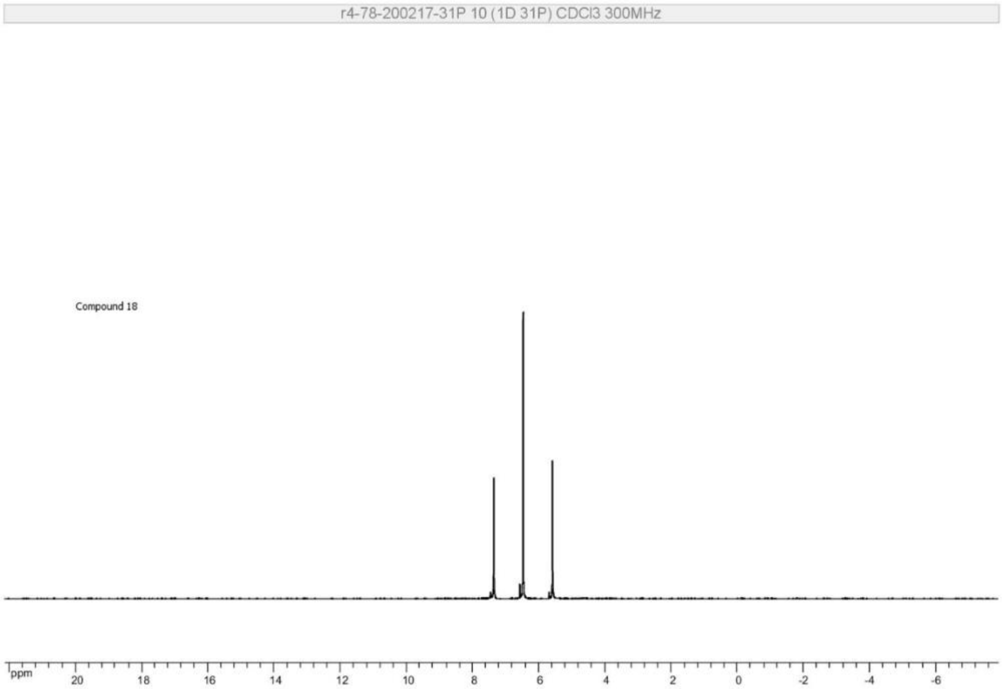
r4-78-200217-31P 10 (1D 31P) CDCl3 300MHz
Compound 18
ppm
20
18
16
14
12
10
8
6
4
2
0
-2
-4
-6

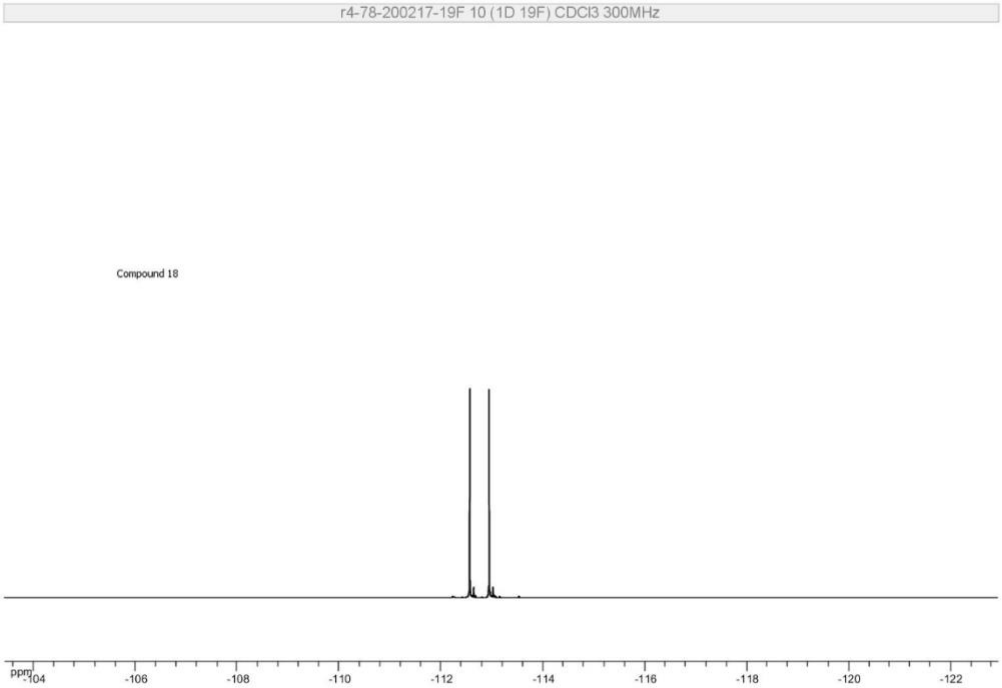
r4-78-200217-19F 10 (1D 19F) CDCl3 300MHz
Compound 18
ppm
-104
-106
-108
-110
-112
-114
-116
-118
-120
-122

Compond **19a**

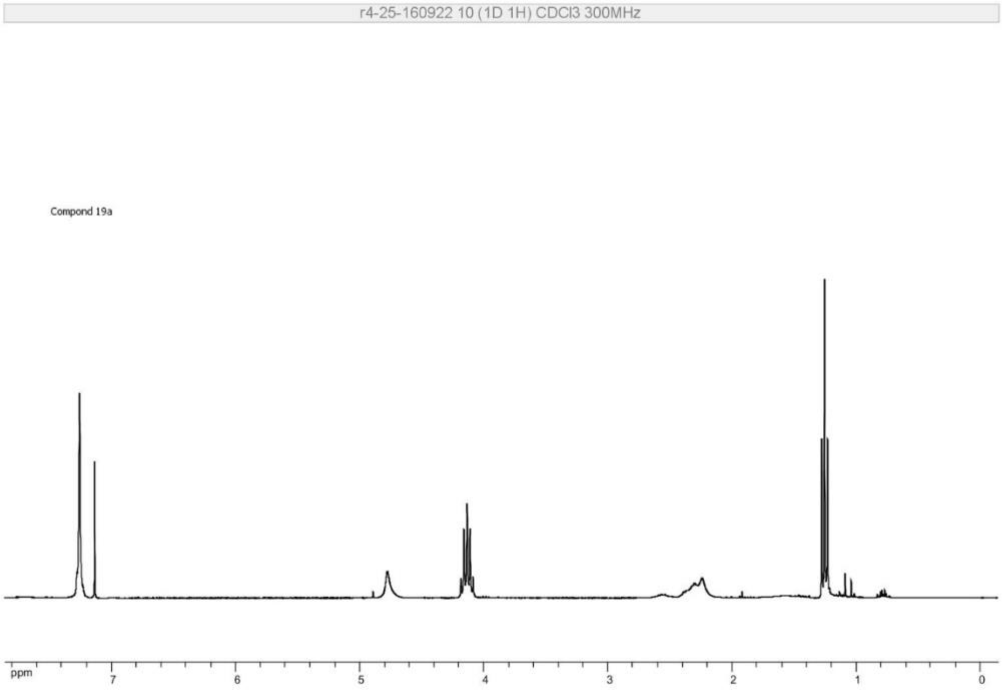

r4-25-160922 10 (1D 1H) CDCl3 300MHz
Compond 19a
ppm
7
6
5
4
3
2
1
0

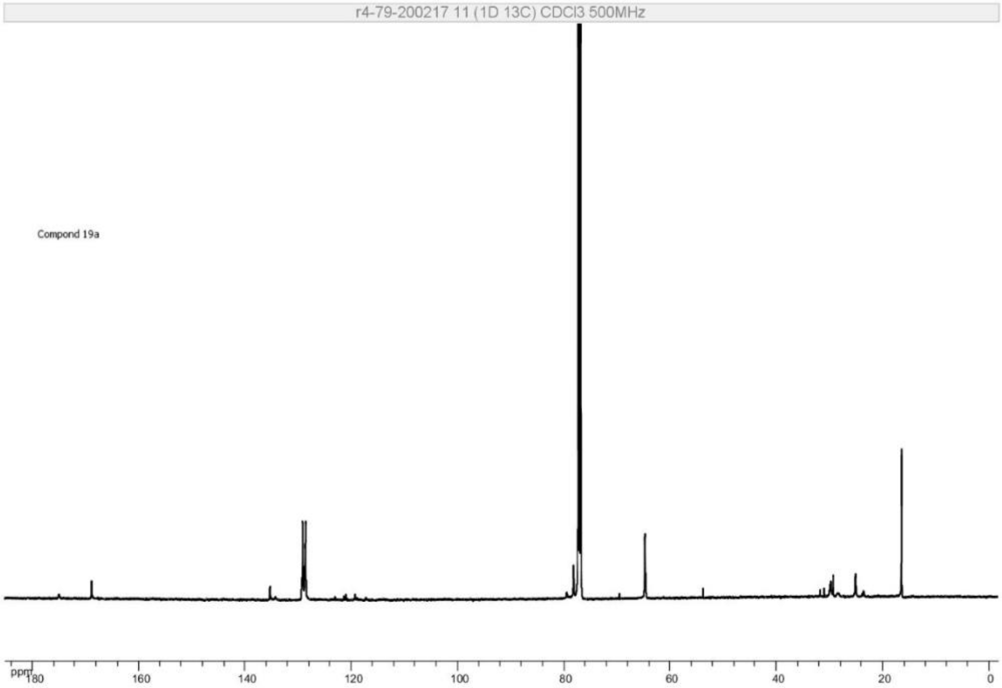

r4-79-200217 11 (1D 13C) CDCl3 500MHz
Compond 19a
ppm
180
160
140
120
100
80
60
40
20
0

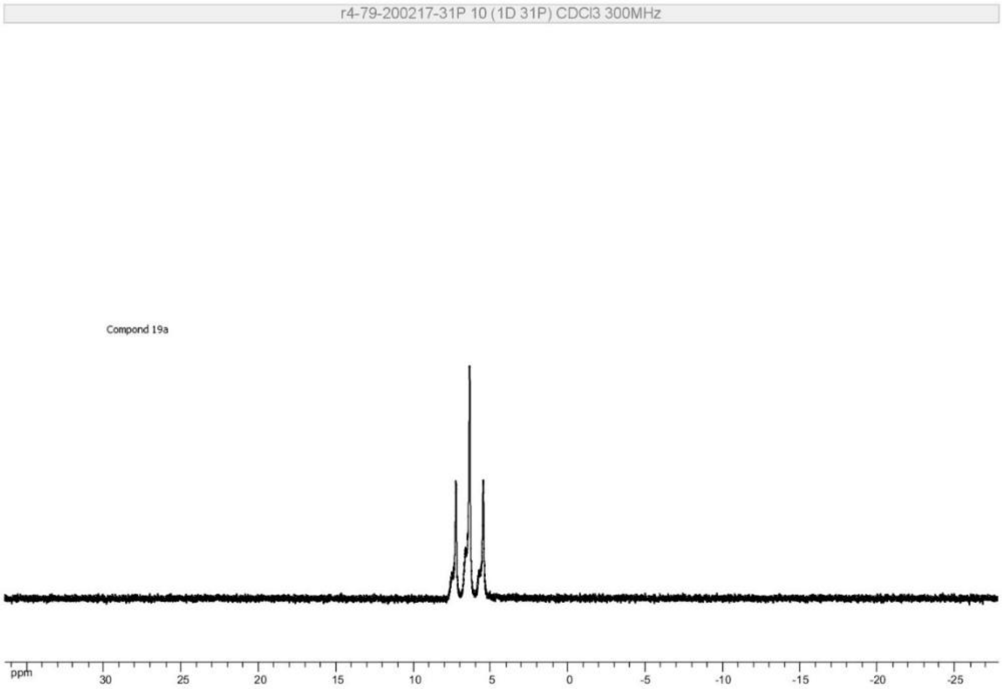
r4-79-200217-31P 10 (1D 31P) CDCl3 300MHz
Compond 19a
ppm
30
25
20
15
10
5
0
-5
-10
-15
-20
-25

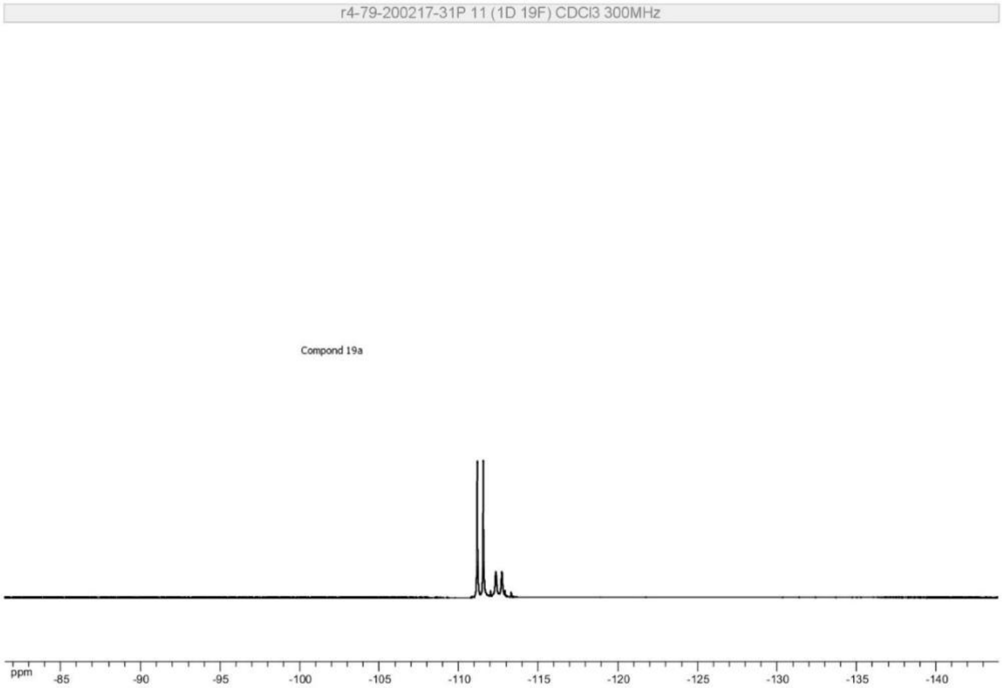
r4-79-200217-31P 11 (1D 19F) CDCl3 300MHz
Compond 19a
ppm
-85
-90
-95
-100
-105
-110
-115
-120
-125
-130
-135
-140

Compound **19b**

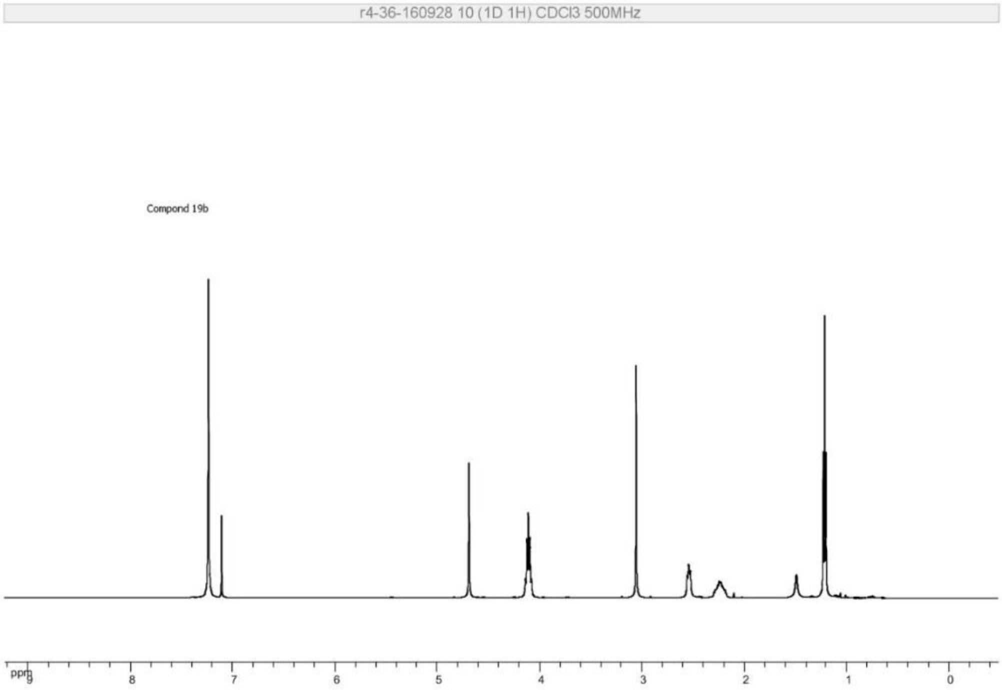

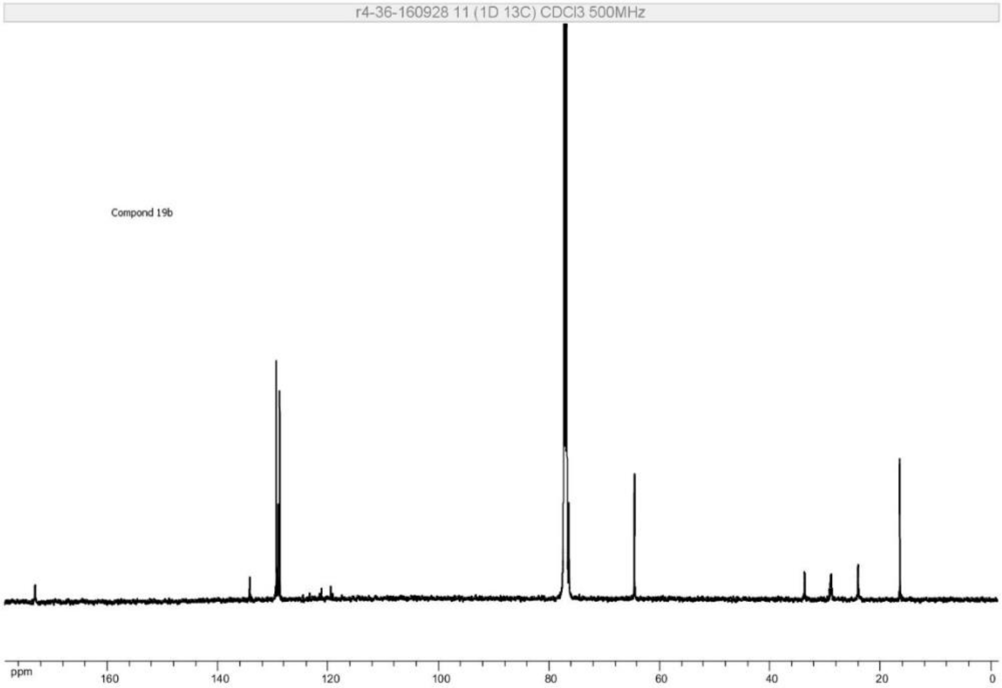

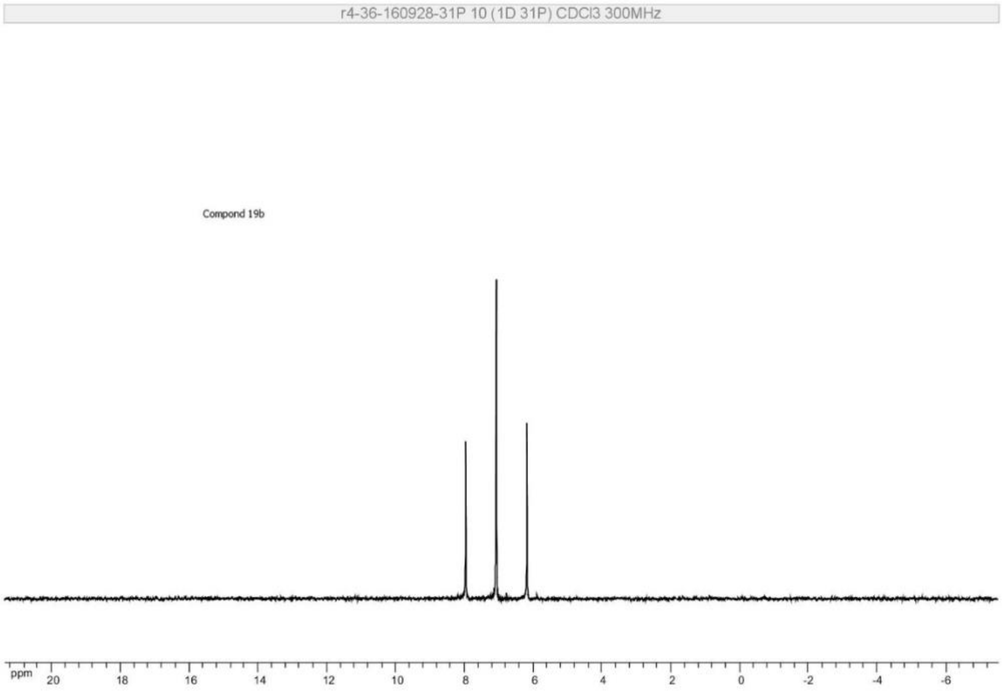

r4-36-160928-31P 10 (1D 31P) CDCl3 300MHz
Compond 19b
ppm
20
18
16
14
12
10
8
6
4
2
0
-2
-4
-6

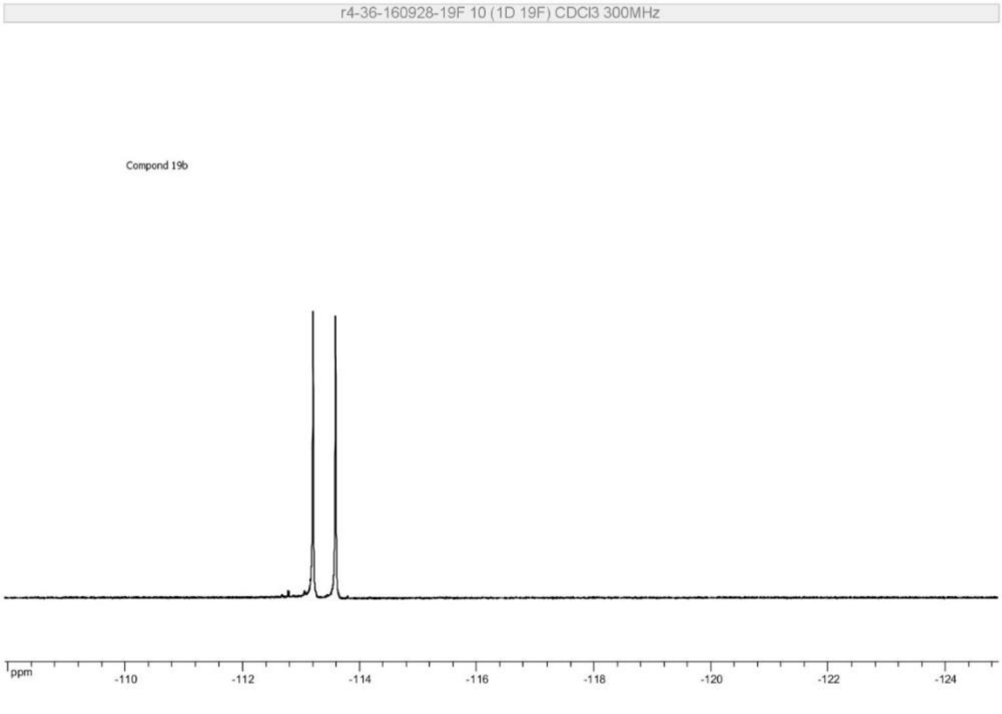

r4-36-160928-19F 10 (1D 19F) CDCl3 300MHz
Compond 19b
ppm
-110
-112
-114
-116
-118
-120
-122
-124

Compound **20a**

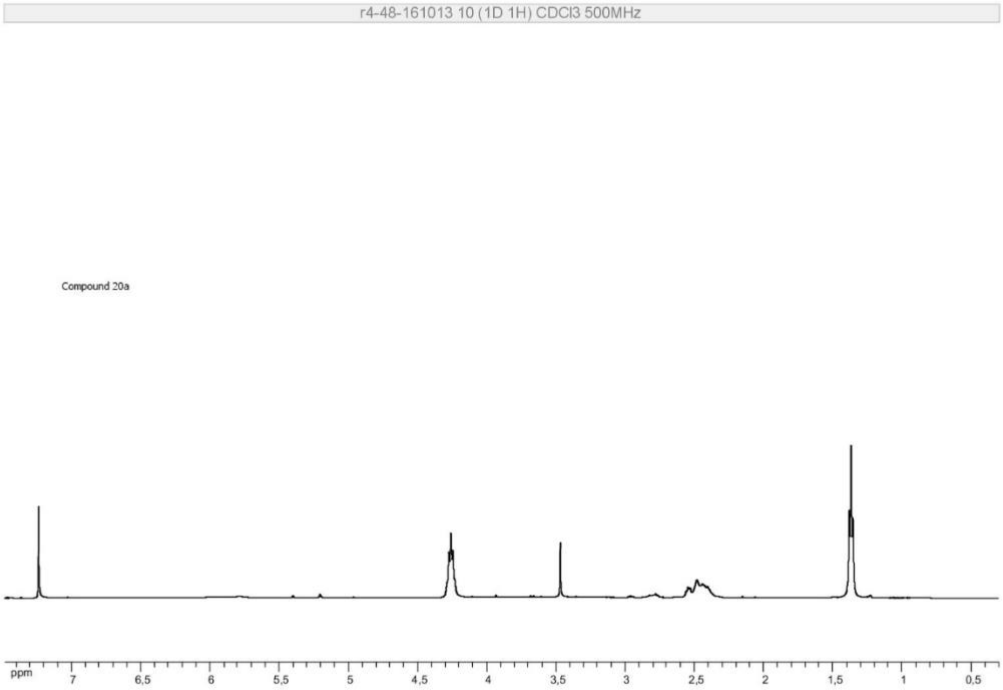

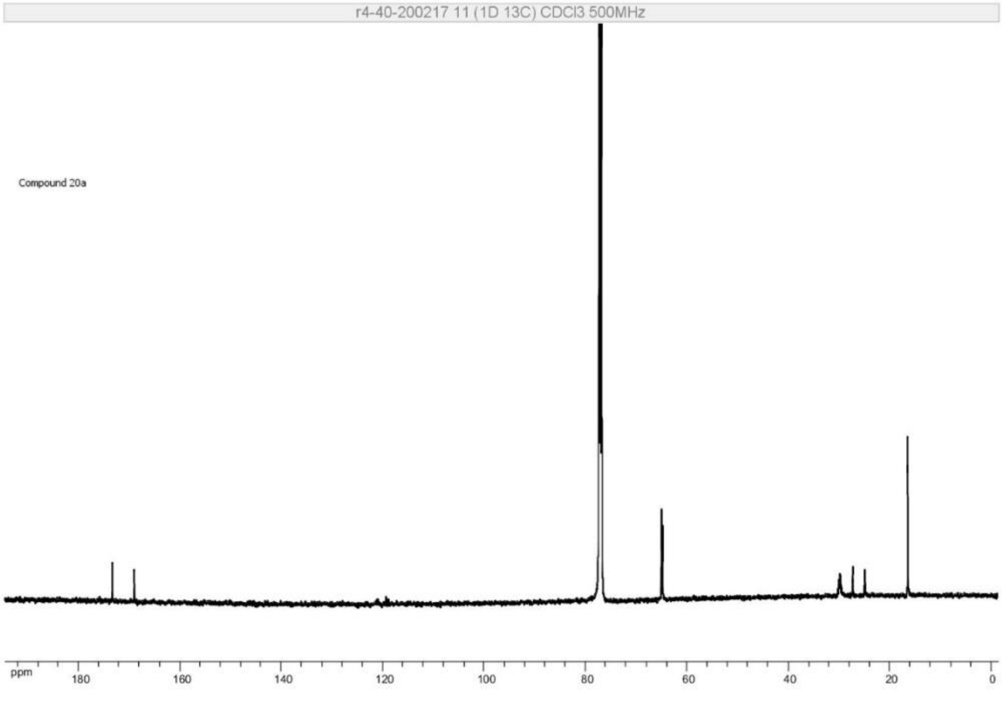

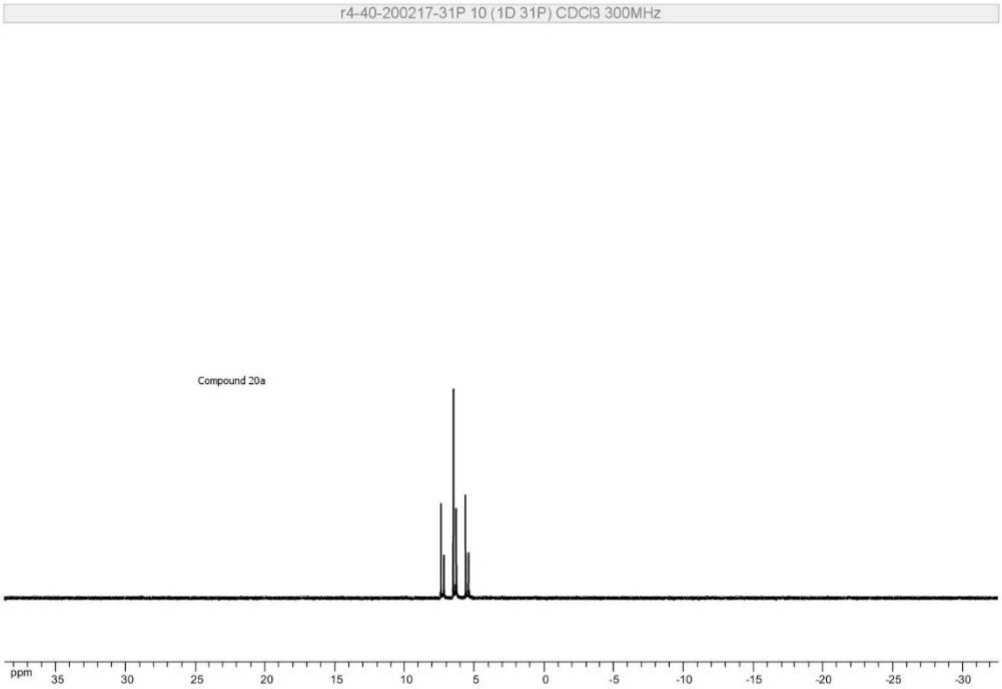
r4-40-200217-31P 10 (1D 31P) CDCl3 300MHz
Compound 20a
ppm
35
30
25
20
15
10
5
0
-5
-10
-15
-20
-25
-30

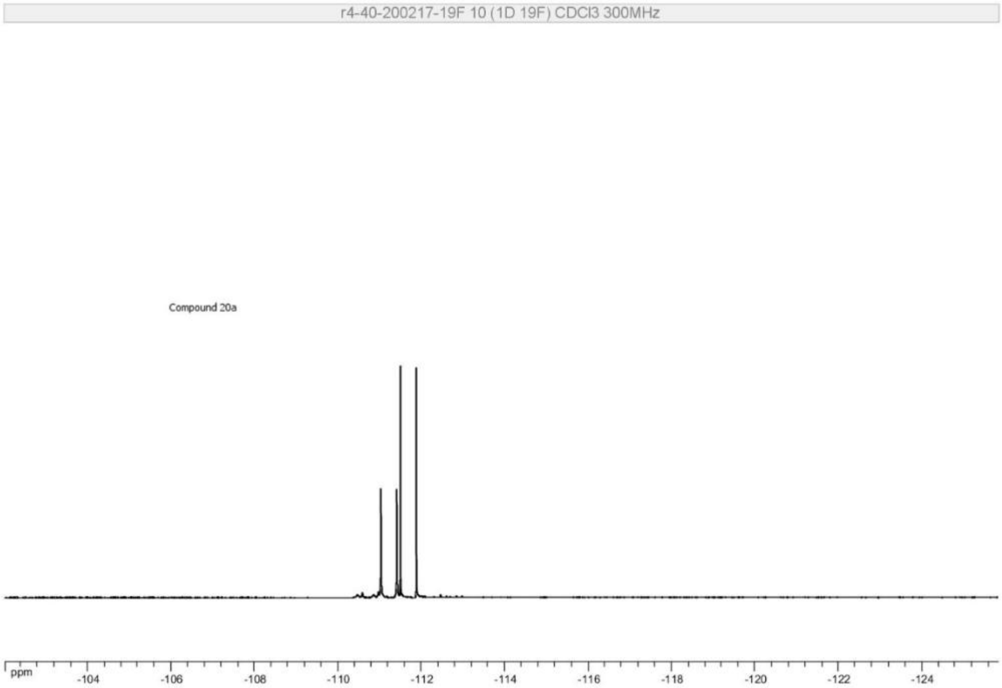
r4-40-200217-19F 10 (1D 19F) CDCl3 300MHz
Compound 20a
ppm
-104
-106
-108
-110
-112
-114
-116
-118
-120
-122
-124

Compound **20b**

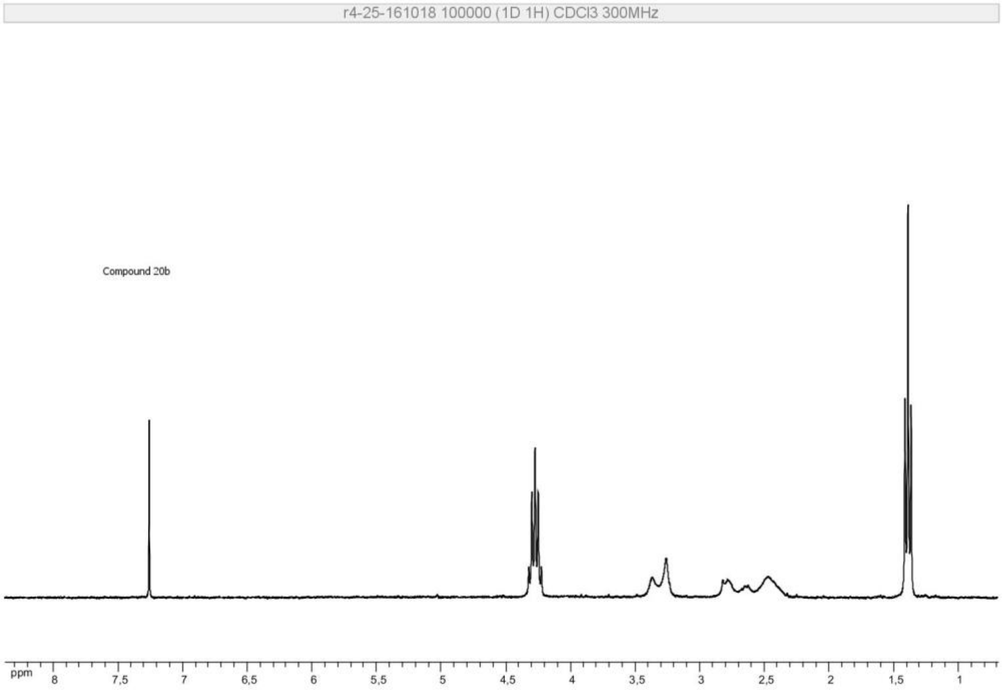

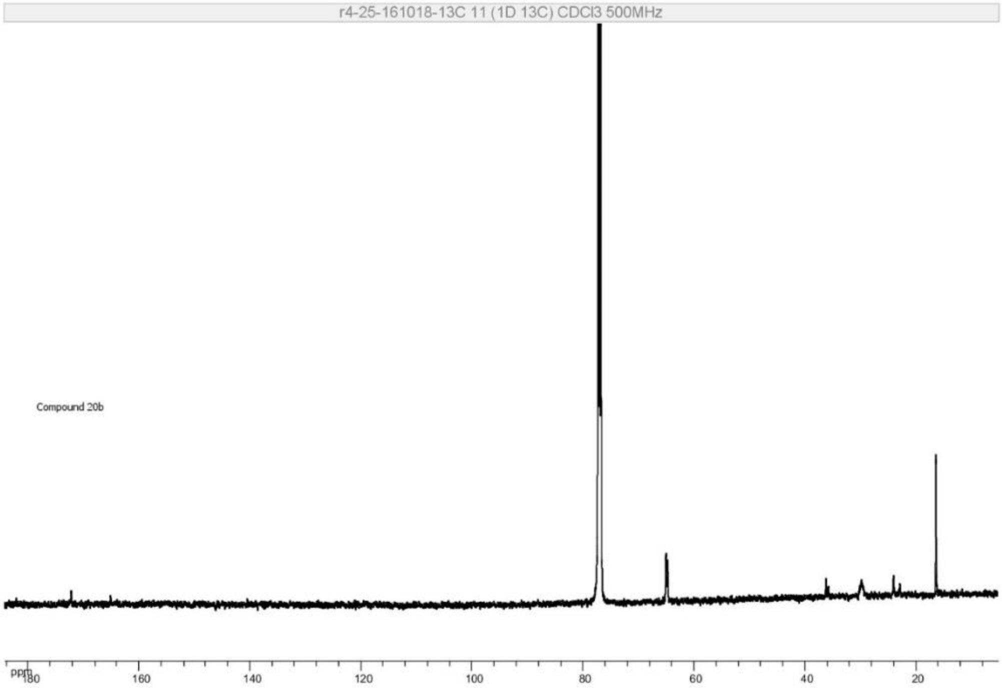

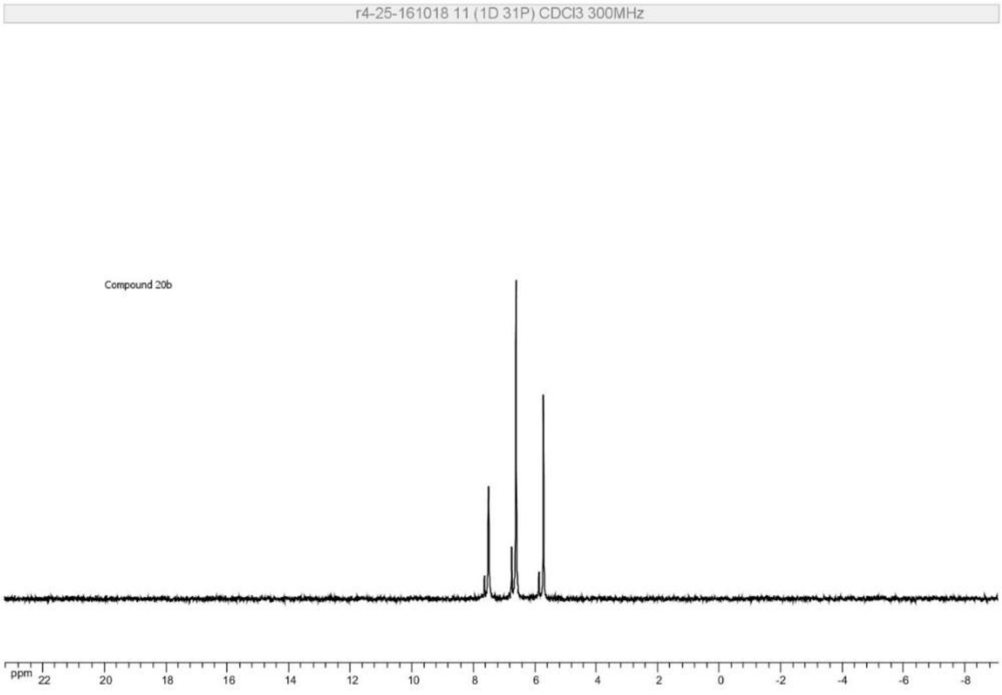
r4-25-161018 11 (1D 31P) CDCl3 300MHz
Compound 20b
ppm 22 20 18 16 14 12 10 8 6 4 2 0 -2 -4 -6 -8

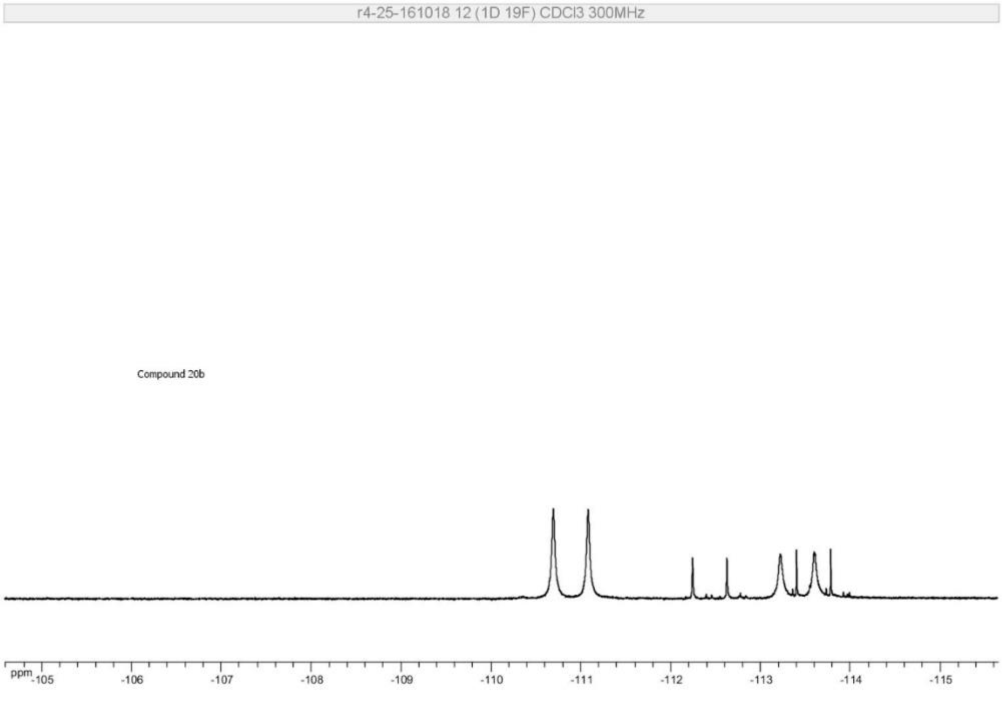
r4-25-161018 12 (1D 19F) CDCl3 300MHz
Compound 20b
ppm -105 -106 -107 -108 -109 -110 -111 -112 -113 -114 -115

Compound **9b**

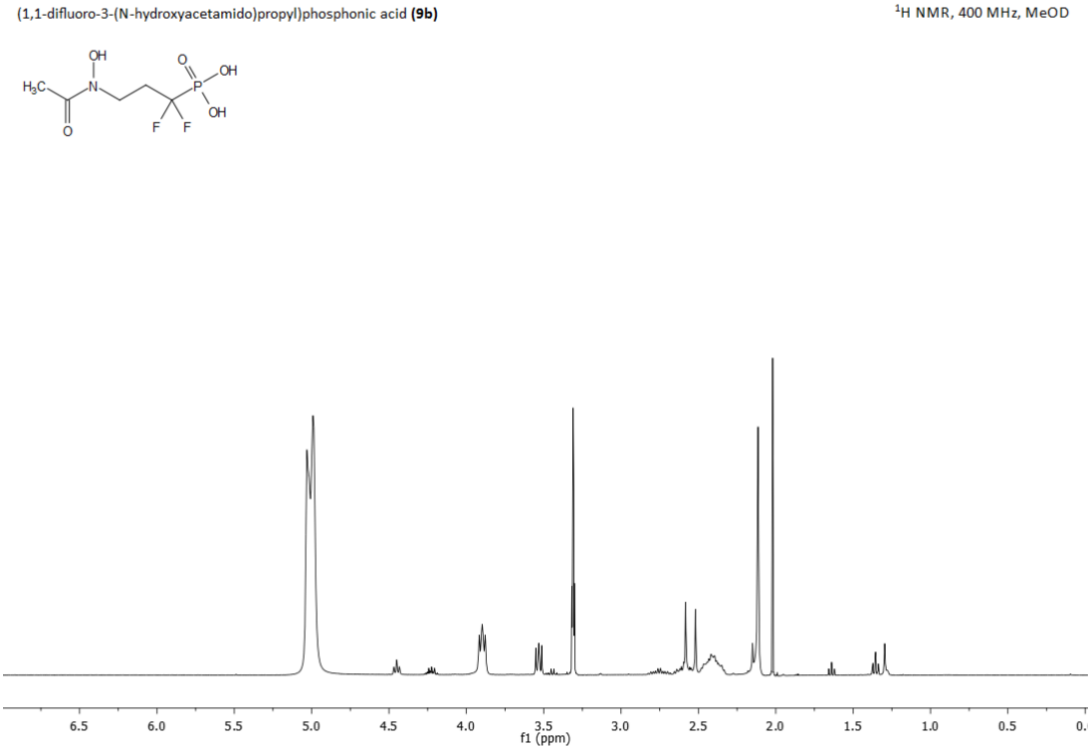

(1,1-difluoro-3-(N-hydroxyacetamido)propyl)phosphonic acid (9b)
1H NMR, 400 MHz, MeOD
f1 (ppm)

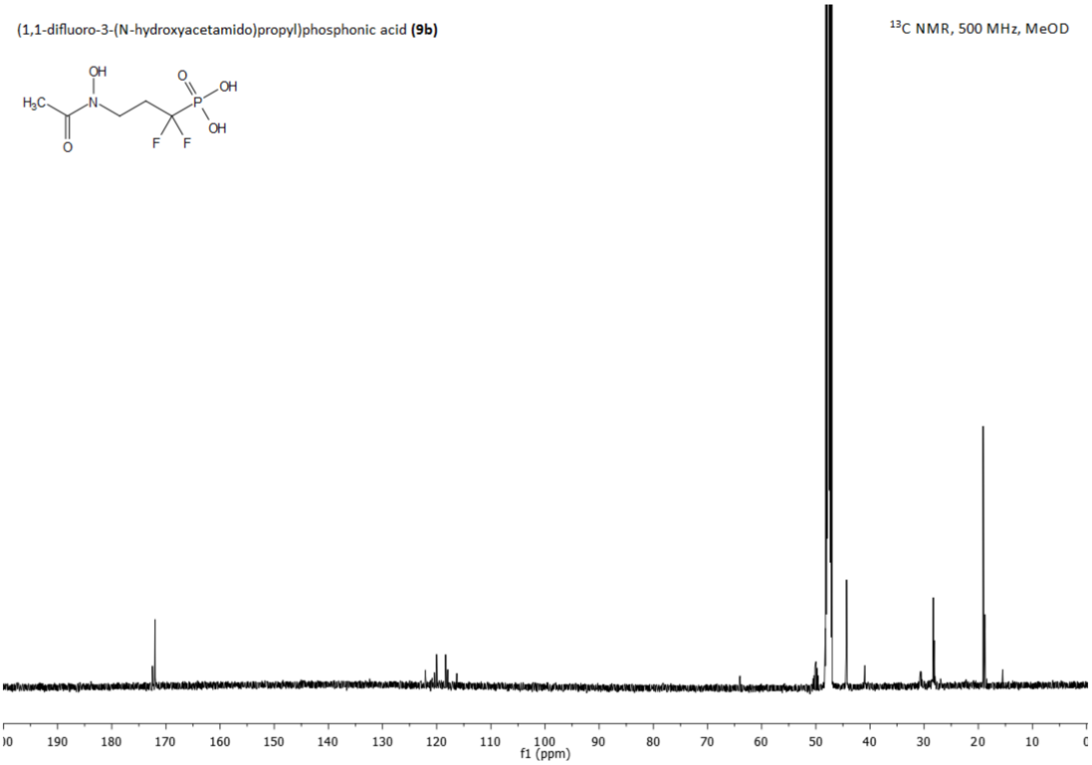

(1,1-difluoro-3-(N-hydroxyacetamido)propyl)phosphonic acid (9b)
13C NMR, 500 MHz, MeOD
f1 (ppm)

(1,1-difluoro-3-(N-hydroxyacetamido)propyl)phosphonic acid **(9b)**

^{19}F NMR, 282 MHz, MeOD

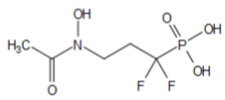

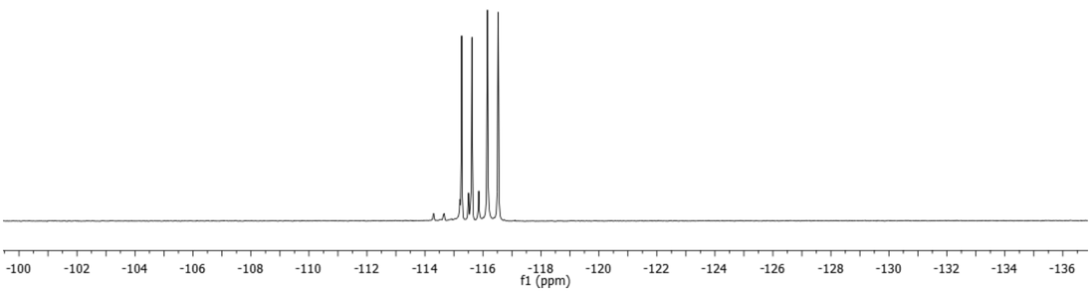

(1,1-difluoro-3-(N-hydroxyacetamido)propyl)phosphonic acid **(9b)**

^{31}P NMR, 162 MHz, MeOD

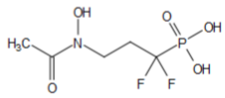

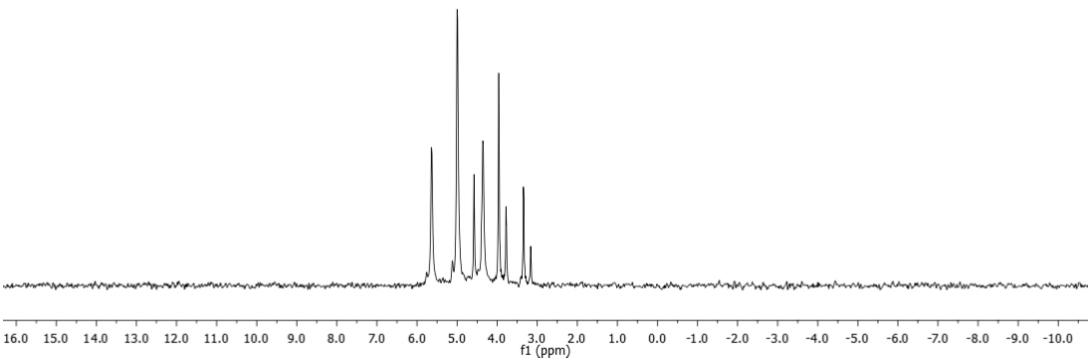

Compound **10a**

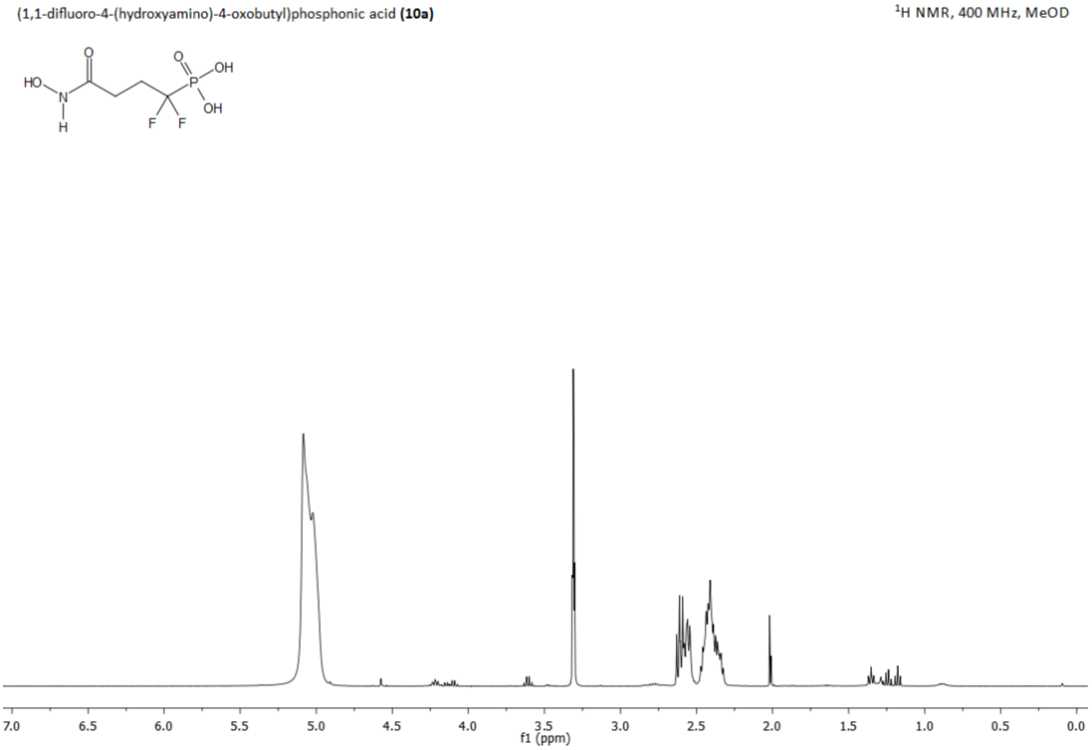

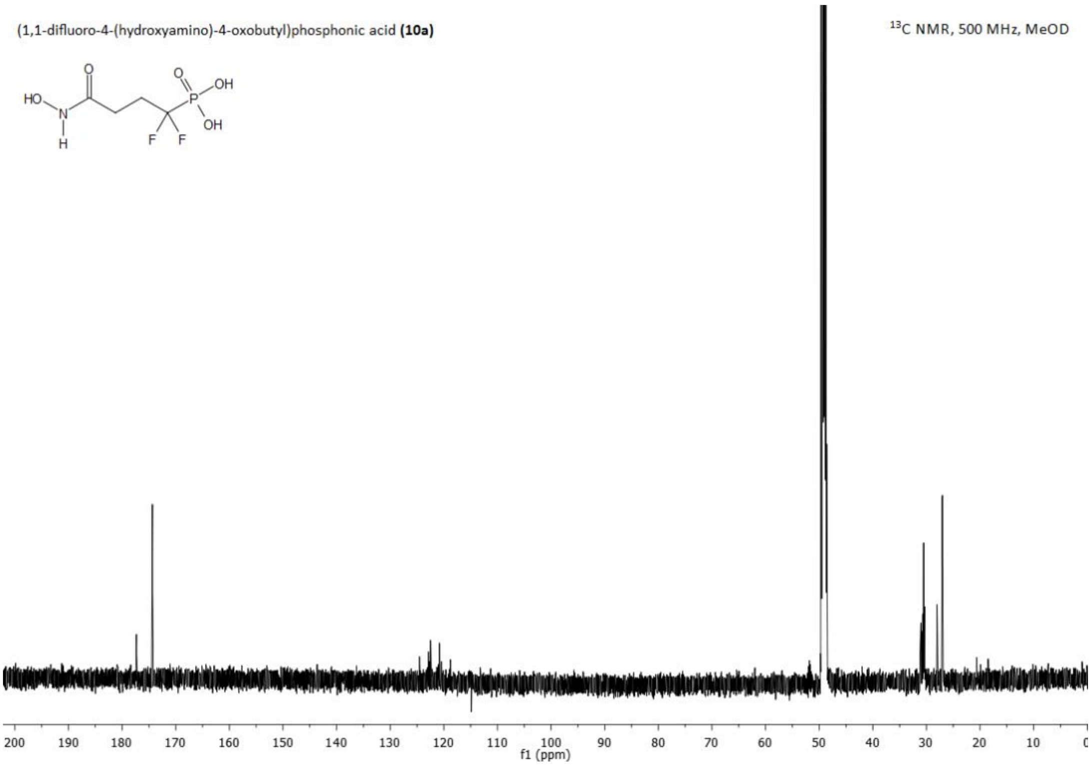

(1,1-difluoro-4-(hydroxyamino)-4-oxobutyl)phosphonic acid **(10a)**

^{19}F NMR, 282 MHz, MeOD

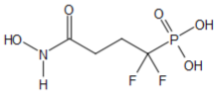

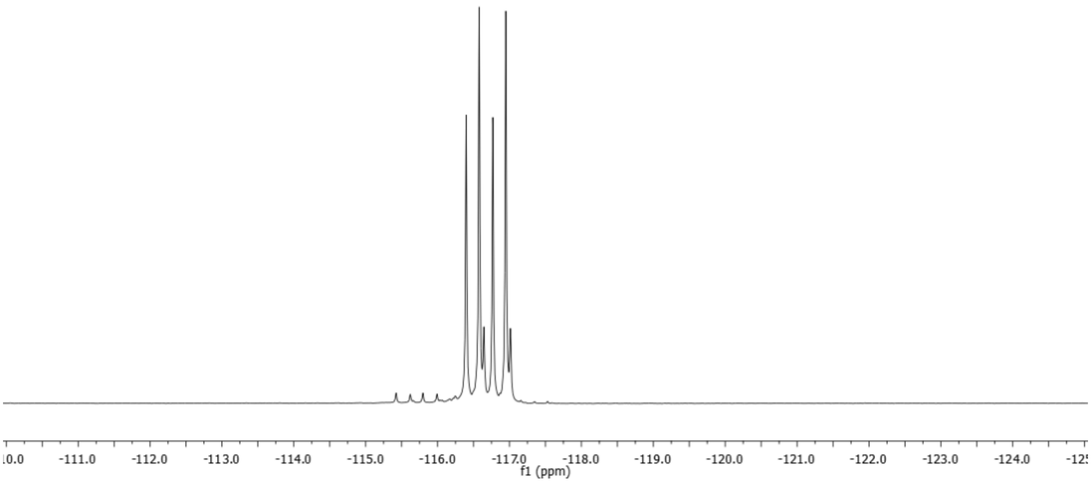

(1,1-difluoro-4-(hydroxyamino)-4-oxobutyl)phosphonic acid **(10a)**

^{31}P NMR, 162 MHz, MeOD

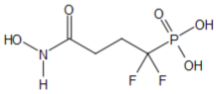

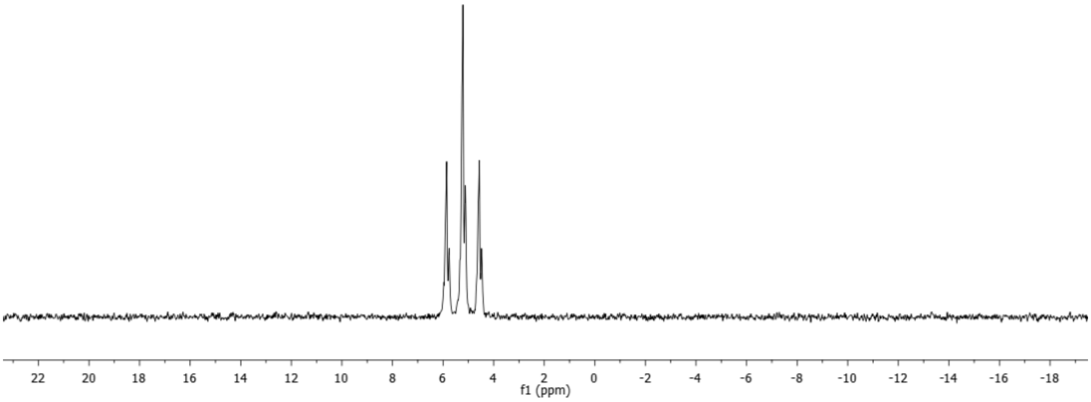

Compound **10b**

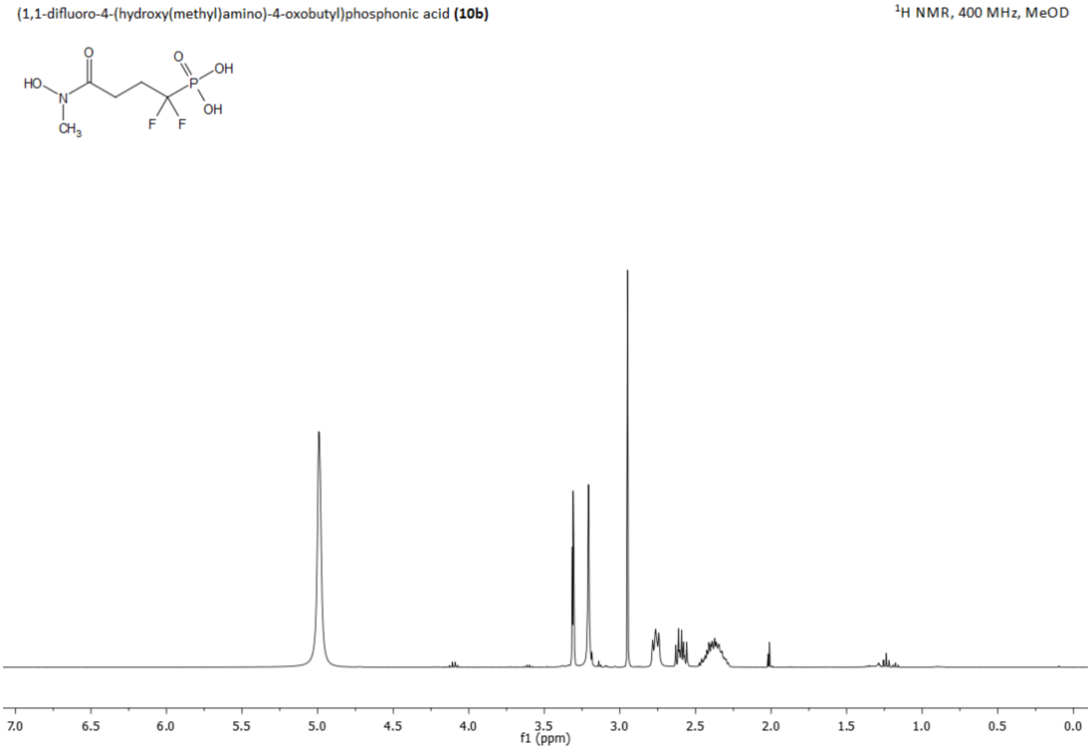

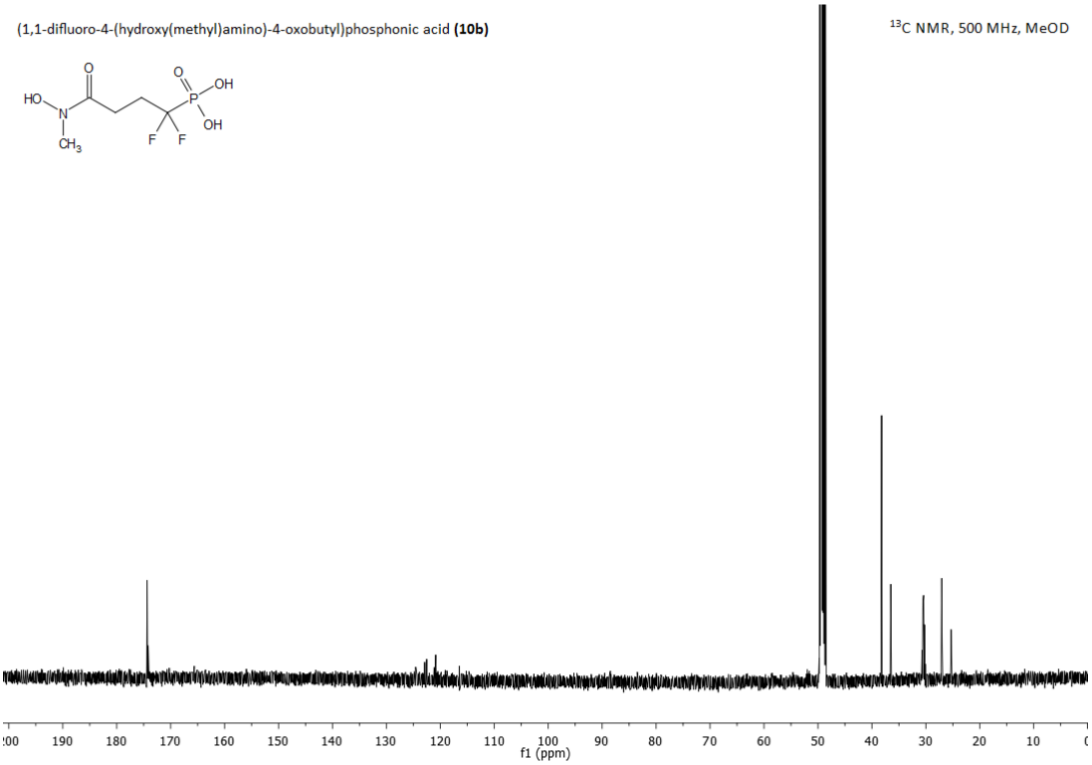

(1,1-difluoro-4-(hydroxy(methyl)amino)-4-oxobutyl)phosphonic acid **(10b)**

^{19}F NMR, 282 MHz, MeOD

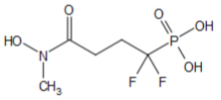

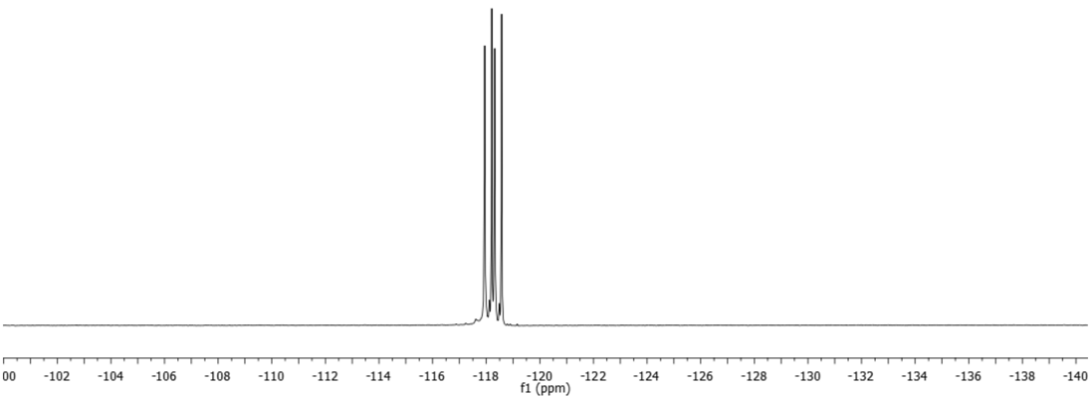

(1,1-difluoro-4-(hydroxy(methyl)amino)-4-oxobutyl)phosphonic acid **(10b)**

^{31}P NMR, 162 MHz, MeOD

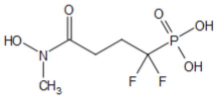

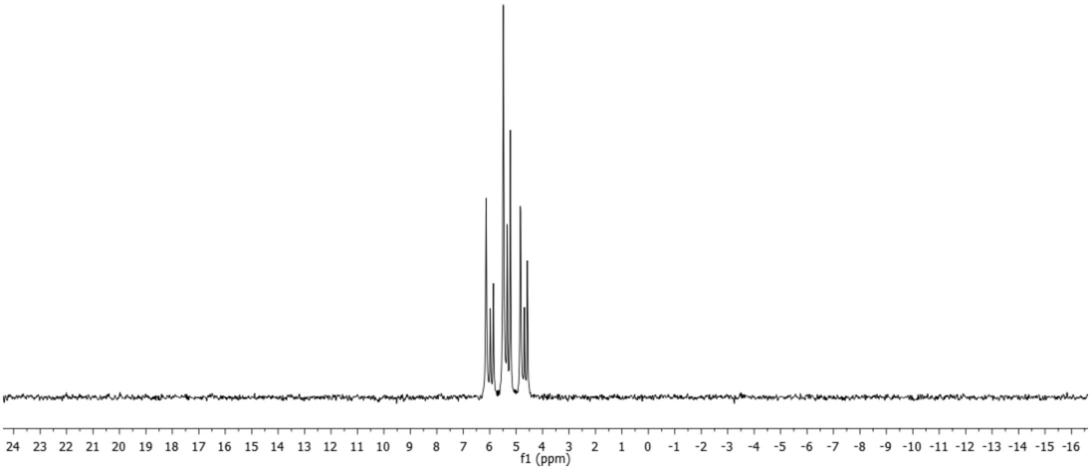

Supplement: Supplementary file 1 [file molecules-26-05111-s001.zip › molecules-1338195-supplementary.pdf]
